# Dithranol targets keratinocytes, their crosstalk with neutrophils and inhibits the IL-36 inflammatory loop in psoriasis

**DOI:** 10.7554/eLife.56991

**Published:** 2020-06-02

**Authors:** Theresa Benezeder, Clemens Painsi, VijayKumar Patra, Saptaswa Dey, Martin Holcmann, Bernhard Lange-Asschenfeldt, Maria Sibilia, Peter Wolf

**Affiliations:** 1 Department of Dermatology, Medical University of GrazGrazAustria; 2 State Hospital KlagenfurtKlagenfurt am WörtherseeAustria; 3 Institute of Cancer Research, Department of Medicine I, Comprehensive Cancer Center, Medical University of ViennaViennaAustria; Radboud University Medical CentreNetherlands; Christian-Albrechts-Universität zu KielKielGermany

**Keywords:** psoriasis, dithranol, anthralin, keratinocytes, AMPs, IL-36, Human, Mouse

## Abstract

Despite the introduction of biologics, topical dithranol (anthralin) has remained one of the most effective anti-psoriatic agents. Serial biopsies from human psoriatic lesions and both the c-Jun/JunB and imiquimod psoriasis mouse model allowed us to study the therapeutic mechanism of this drug. Top differentially expressed genes in the early response to dithranol belonged to keratinocyte and epidermal differentiation pathways and IL-1 family members (i.e. *IL36RN)* but not elements of the IL-17/IL-23 axis. In human psoriatic response to dithranol, rapid decrease in expression of keratinocyte differentiation regulators (e.g. involucrin, *SERPINB7* and *SERPINB13*), antimicrobial peptides (e.g. ß-defensins like *DEFB4A, DEFB4B, DEFB103A,* S100 proteins like *S100A7, S100A12*), chemotactic factors for neutrophils (e.g. *CXCL5, CXCL8*) and neutrophilic infiltration was followed with much delay by reduction in T cell infiltration. Targeting keratinocytes rather than immune cells may be an alternative approach in particular for topical anti-psoriatic treatment, an area with high need for new drugs.

## Introduction

With the development and market introduction of biologics, much progress has been made in recent years in the systemic treatment of psoriasis. Currently, targeted therapy with antibodies against IL-17 or IL-23 exhibits high efficacy and allows complete or almost complete clinical clearance of psoriasis lesions in a high percentage of cases (Blauvelt et al., 2017; Langley et al., 2014; Papp et al., 2017; Reich et al., 2018). However, patients with limited body area involvement (i.e. mild forms of psoriasis) who represent the majority of psoriasis patients with up to 90% (Stern et al., 2004) have been neglected. Not much innovation has occurred in the past few years on topical treatment of psoriasis that is mainly prescribed to these patients. Steroids, vitamin D3 and vitamin A analogues are most commonly used as topical agents in such patients, but besides changes in their pharmaceutical formulation, they have not been developed further in recent years (de Korte et al., 2008; Krueger et al., 2001; Langley et al., 2011; Schaarschmidt et al., 2015). Short courses of dithranol (1,8-dihydroxy-9-anthracenone or anthralin) have been successfully used as intermittent topical treatment of psoriasis since 1916 (Nast et al., 2012; Sehgal et al., 2014; Vleuten et al., 1996). Despite its numerous disadvantages like brown staining and irritation of perilesional skin, dithranol has remained one of the most effective topical treatment modalities in psoriasis. Analogous to the most recent generation of biologics (including anti-IL-17 and anti-IL-23 antibodies), dithranol delivers PASI75 rates in 66–82.5% of patients with fast action and clearance of skin lesion within very few weeks (Kemény et al., 1990; Painsi et al., 2015b; Sehgal et al., 2014; van de Kerkhof, 2015). Although dithranol has been used for many years, its exact mechanism of action has remained largely unknown (Holstein et al., 2017; Kemény et al., 1990; Kucharekova et al., 2006; Sehgal et al., 2014).

With the aim of unraveling dithranol’s therapeutic mechanisms and to possibly uncover new targets for topical treatment of psoriasis, we conducted a clinical trial and employed several mouse models including the c-Jun/JunB knockout model (Zenz et al., 2005) and the imiquimod psoriasis model (van der Fits et al., 2009) in order to address this issue and elucidate dithranol's effects. In this study, we demonstrate that dithranol exerts its anti-psoriatic effects by directly targeting keratinocytes and their crosstalk with neutrophils, as well as disrupting the IL-36 inflammatory loop. Consistent with this finding, we observed that dithranol’s therapeutic activity was completely independent of its pro-inflammatory effect mainly on perilesional skin, thus overthrowing the long-believed paradigm that dithranol-induced irritation is crucial for its anti-psoriatic action and unraveling irritation merely as a bystander effect of treatment.

## Results

### Topical dithranol leads to fast reduction in PASI score linked to decrease in epidermal hyperproliferation and delayed reduction of inflammatory infiltrate in psoriatic skin

**Figure 1.**
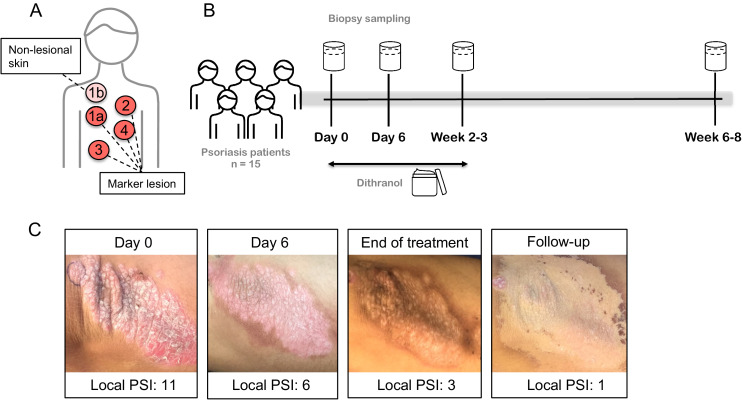
Dithranol leads to a fast reduction of psoriatic skin lesions as determined by local psoriasis severity index (PSI) of marker lesions. (**A,B**) 15 psoriasis patients were treated with dithranol. Skin biopsies were taken from marker lesions at multiple timepoints: 1a = lesional skin at baseline, 2 = lesional skin at day 6, 3 = lesional skin at end of treatment, week 2–3, 4 = lesional skin at follow-up, 4–6 weeks after end of treatment. In addition, non-lesional skin at baseline (1b) was sampled. (**C**) Representative images of lesional psoriatic skin and local psoriasis severity index (PSI; sum of erythema (0–4), induration (0–4) and scaling (0–4); 0 = none, 1 = mild, 2 = moderate, 3 = severe, 4 = very severe) at different time points.

**Figure 2.**
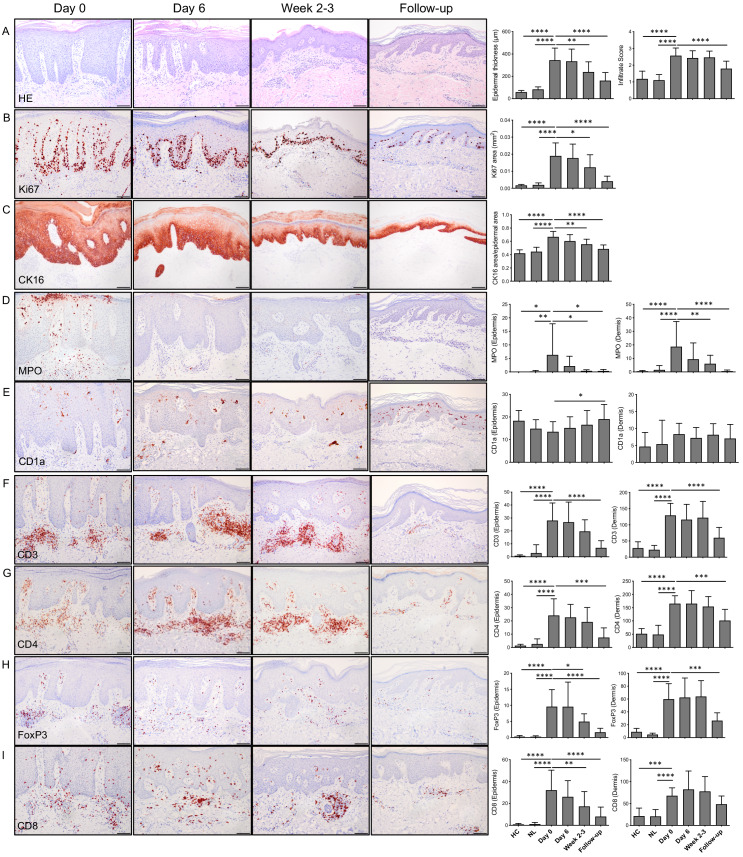
Histological and immunohistochemical analysis of lesional skin before (day 0), during (day 6), at end of treatment (week 2–3) and 4–6 weeks after ending treatment (follow-up). (**A**) Representative H and E images, epidermal thickness and cellular infiltrate scoring. In treated psoriatic lesions, epidermal thickness was significantly decreased at week 2–3 and at follow-up. Infiltrate score (0 = none, 0.5 = none/low, 1 = low, 1.5 = low/moderate, 2 = moderate, 2.5 = moderate/high, 3 = high infiltration of immune cells) was significantly higher in untreated psoriatic lesions compared to non-lesional skin (NL) and healthy controls (HC). In treated psoriatic lesions, infiltrate score was significantly decreased at follow-up. (**B**) Ki67 staining in epidermis was significantly reduced at week 2–3 and follow-up. (**C**) CK16 staining in epidermis was significantly reduced at week 2–3 and follow-up. (**D–I**) Representative IHC images and mean cell counts for epidermis and dermis. Neutrophil cell counts were significantly reduced at week 2–3 and follow-up in epidermis and dermis (**D**). Langerhans cell (CD1a+) numbers in epidermis were significantly increased at follow-up (**E**). Epidermal FoxP3 (**H**) and CD8 (**I**) cell counts were significantly reduced at week 2–3. Dermal CD3 (**F**), CD4 (**G**), and FoxP3 (**H**) positive cell counts display significant reduction at follow-up. One-way ANOVA with Dunnet’s multiple comparisons test was used for statistics. Bars represent mean ± SD; *p≤0.05; **p≤0.01; ***p≤0.001; ****p≤0.0001; scale bar = 100 µm. Figure 2—source data 1.Values displayed in bar plots shown in Figure 2.

As depicted in Figure 1C (and Figure 1—figure supplement 1), dithranol did lead to a fast reduction of psoriatic skin lesions in most of the 15 patients of the study, as determined by psoriasis area and severity index (PASI) and local psoriasis severity index (PSI) of marker lesions. As shown in Table 1, the mean decrease in PASI score was 58% after 2–3 weeks, confirming its high clinical efficacy (Painsi et al., 2015a; Painsi et al., 2015b; Swinkels et al., 2004). Remarkably, dithranol treatment did lead to a visible inflammatory response only at perilesional skin sites, but not within psoriatic plaques and there was no correlation between dithranol-induced erythema and its anti-psoriatic effect (Figure 1—figure supplement 2). The clinical response was confirmed by the results of histological analysis of skin biopsies taken throughout the dithranol treatment course (Figure 1A and B). Dithranol application led to a significant reduction in epidermal hyperplasia (as measured by thickness of epidermis). Intriguingly, there was no significant change in dermal infiltrate score during treatment. At the follow-up visit, hyperplasia of the epidermis was reduced further and cellular infiltrate in the dermis was significantly diminished (Figure 2).

**Figure 1—figure supplement 1.**
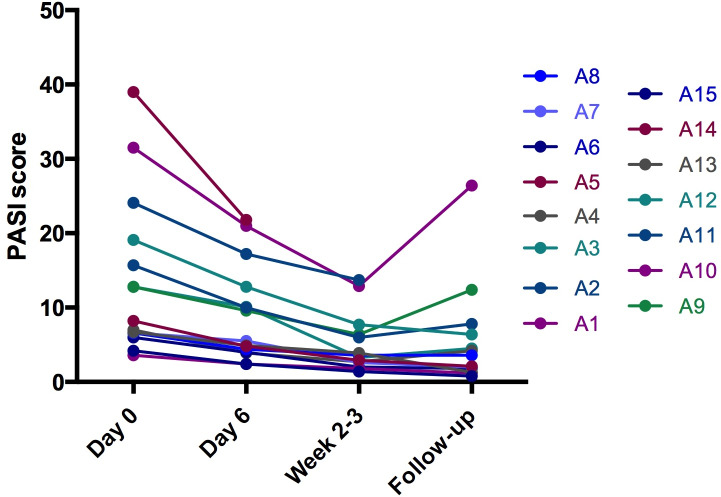
Psoriasis area and severity (PASI) score at baseline (day 0), early during treatment (day 6), at end of treatment (week 2–3) and at follow-up (4–6 weeks after end of treatment) for all 15 patients (A1–A15) treated with dithranol. Figure 1—figure supplement 1—source data 1.Values displayed in graph shown in Figure 1—figure supplement 1.

**Figure 1—figure supplement 2.**
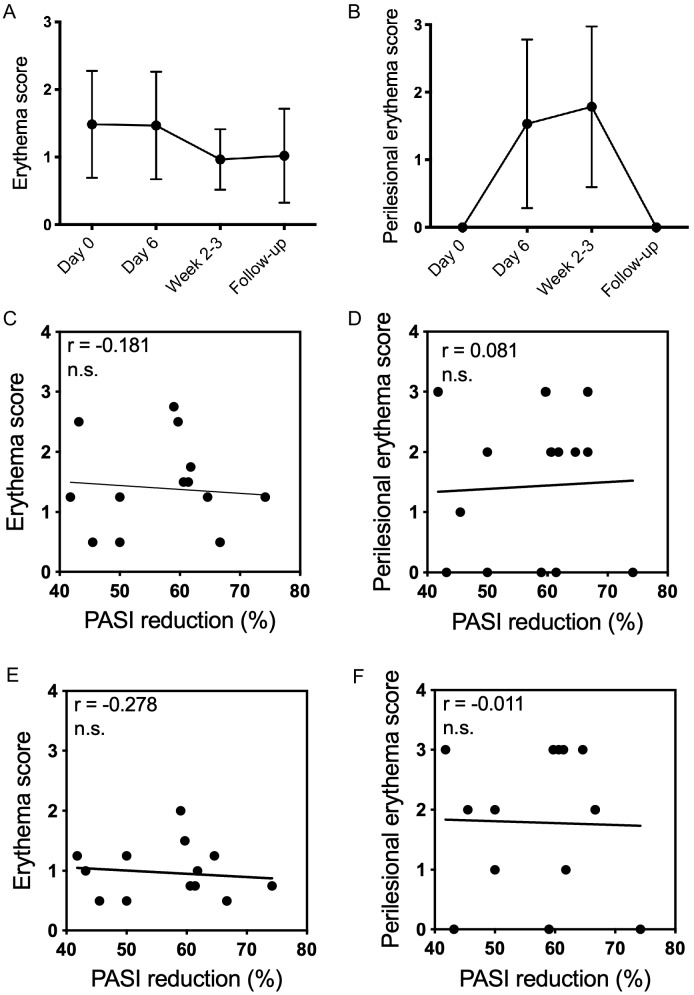
Lesional and perilesional erythema does not correlate with reduction in PASI score. (**A**) Mean erythema score (0 = none, 1 = mild, 2 = moderate, 3 = severe 4 = very severe erythema on lesional skin) and B) Mean perilesional erythema score (0 = none, 1 = mild, 2 = moderate, 3 = severe erythema on perilesional skin) at baseline (day 0), early during dithranol treatment (day 6), at end of treatment (week 2–3) and at follow-up (4–6 weeks after end of treatment). Mean ± SD (n = 15) is shown. (**C–D**) Erythema score (**C**) and perilesional erythema score (**D**) at day six during dithranol treatment was compared with reduction in PASI score at end of treatment using Spearman correlation. (**E–F**) Erythema score (**E**) and perilesional erythema score (**F**) at end of treatment (week 2–3) was compared with reduction in PASI score at end of treatment using Spearman correlation. n.s. = not significant.

**Table 1.** Psoriasis area and severity index (PASI) and psoriasis severity index (PSI) from 15 psoriasis patients. Table 1—source data 1.Values displayed in Table 1.

Parameter		Time point			
		Baseline	Day 6	End of treatment	Follow-up
PASI					
	mean ± SD	13.6 ± 10.3	9.0 ± 6.3 (p<0.0001)^*^	5.1 ± 3.8 (p=0.0001)^*^	5.7 ± 6.7 (p=0.0002)^*^
	%reduction, mean ± SD (range)	-	32.9 ± 8.0 (16.7–44.3)	57.5 ± 9.5 (41.8–74.2)	56.1 ± 23.3 (3.1–81.0)
Local PSI					
	mean ± SD	6.7 ± 1.1	3.3 ± 1.6 (p<0.0001)^*^	2.0 ± 1.5 (p<0.0001)^*^	1.6 ± 1.3 (p<0.0001)^*^
	%reduction, mean ± SD (range)	-	52.6 ± 21.9 (14.3–100)	69.0 ± 23.9 (16.7–100)	76.3 ± 18.3 (37.5–100)
					

^*^P value was determined using Wilcoxon test comparing indicated value to baseline.

### Clinical response to dithranol in psoriasis patients is linked to I) fast upregulation of keratinization genes and downregulation of neutrophil chemotactic genes and neutrophilic infiltration followed by II) delayed downregulation of inflammatory response-related genes and proteins

Dithranol slowly diminished CD3, CD4, FoxP3 and CD8 cell counts in the dermis as evidenced by unaffected cell numbers early on during the treatment course and significant reduction only at the follow-up visit (4–6 weeks after end of treatment) (Figure 2). The response of T cells in the epidermis occurred a little earlier, with significant reduction present already at the end of treatment (week 2–3) (Figure 2). In agreement with other studies (Holstein et al., 2017; Swinkels et al., 2002a; Vleuten et al., 1996; Yamamoto and Nishioka, 2003), we found a fast decrease in epidermal hyperproliferation (epidermal thickness, Ki-67 and CK16 staining) at end of treatment (week 2–3). Neutrophil numbers (as assessed by myeloperoxidase staining) in epidermis and dermis were also significantly reduced at that time point. An increase in the number of Langerhans cells in the epidermis (as indicated by CD1a staining) was detected at the follow-up visit.

Microarray analysis comparing lesional skin at day 6 of treatment with lesional skin samples at baseline revealed that dithranol led to differential expression of 62 genes including 17 genes with reduced and 45 genes with increased expression. Among these differentially expressed genes (DEGs), there was a significant upregulation of genes involved in keratinization and keratinocyte differentiation (e.g. *KRT2, LCE1C, KRT73, LCE1A*) and establishment of skin barrier (e.g. *FLG2, HRNR, FLG*). Furthermore, dithranol downregulated genes of antimicrobial peptides (AMPs) such as ß-defensin-2 (*DEFB4A, DEFB4B*) and chemoattractants for neutrophils (e.g. *CXCL5, CXCL8, PPBP1, TREM1*) (Table 2).

**Table 2.** Top 45 differentially regulated genes (p-value<0.05, fold change >1.5) in dithranol-treated lesional skin after 6 days compared to baseline from 15 patients with psoriasis.

Probe set ID	Gene symbol	Gene title	Day 6 vs. baseline (fold change)
16693318	FLG2	Filaggrin family member 2	2.89
16693303	HRNR	Hornerin	2.64
16903552	NEB	Nebulin	2.18
16765017	KRT2	Keratin 2	2.04
16693308	FLG	Filaggrin	1.97
16761221	CLEC2A	C-type lectin domain family 2, member A	1.92
16730782	ELMOD1	ELMO/CED-12 domain containing 1	1.91
16735288	OVCH2	Ovochymase 2	1.83
16990787	SPINK7	Serine peptidase inhibitor, Kazal type 7 (putative)	1.78
16852824	SERPINB12	Serpin peptidase inhibitor, clade B (ovalbumin), member 12	1.74
16693341	LCE1C	Late cornified envelope 1C	1.71
16693249	THEM5	Thioesterase superfamily member 5	1.70
16921644	MIRLET7C	MicroRNA let-7c	1.69
16670681	ANXA9	Annexin A9	1.68
16821186	CLEC3A	C-type lectin domain family 3, member A	1.68
16859090	CASP14	Caspase 14	1.68
16765005	KRT73	Keratin 73	1.66
16945497	COL6A5	Collagen, type VI, alpha 5	1.66
16976615	SULT1E1	Sulfotransferase family 1E, estrogen-preferring, member 1	1.65
16898858	CD207	CD207 molecule, langerin	1.63
16861126	UPK1A	Uroplakin 1A	1.60
16704475	FAM35DP	Family with sequence similarity 35, member A pseudogene	1.60
17085015	FRMPD1	FERM and PDZ domain containing 1	1.59
16817034	CHP2	Calcineurin-like EF-hand protein 2	1.56
16671082	LCE1A	Late cornified envelope 1A	1.55
16671037	LCE2D	Late cornified envelope 2D	1.55
16748835	PIK3C2G	Phosphatidylinositol-4-phosphate 3-kinase, catalytic subunit	1.54
17039517	LY6G6C	Lymphocyte antigen six complex, locus G6C	1.53
16673713	FMO2	Flavin containing monooxygenase 2 (non-functional)	1.52
16812344	BCL2A1	BCL2-related protein A1	−1.52
16968213	ANXA3	Annexin A3	−1.54
17104519	RNY4P23	RNA, Ro-associated Y4 pseudogene 23	−1.55
16948835	MIR1224	MicroRNA 1224	−1.55
16815310	TNFRSF12A	Tumor necrosis factor receptor superfamily, member 12A	−1.60
17015084	SERPINB1	Serpin peptidase inhibitor, clade B (ovalbumin), member 1	−1.67
17106398	SLC6A14	Solute carrier family 6 (amino acid transporter), member 14	−1.69
16693375	SPRR2F	Small proline-rich protein 2F	−1.69
17065458	DEFB4A	Defensin, beta 4A	−1.75
17074361	DEFB4B	Defensin, beta 4B	−1.77
16698947	RNU5A-8P	RNA, U5A small nuclear 8, pseudogene	−1.78
16976827	CXCL5	Chemokine (C-X-C motif) ligand 5	−1.81
16976821	PPBP	Pro-platelet basic protein (chemokine (C-X-C motif) ligand 7)	−1.82
17019056	TREM1	Triggering receptor expressed on myeloid cells 1	−1.99
17050251	SLC26A4	Solute carrier family 26 (anion exchanger), member 4	−2.19
16967771	CXCL8	Chemokine (C-X-C motif) ligand 8	−3.36

At the end of treatment (week 2–3), dithranol had deregulated a total of 453 genes, with downregulation of 325 DEGs and upregulation of 128 genes. Compared to day 6, expression of various genes involved in keratinocyte differentiation decreased further upon dithranol treatment and other AMPs (e.g. *S100A7A, S100A12, DEFB103A*) and genes involved in neutrophil-mediated inflammatory responses significantly diminished in their expression. With delay, dithranol also lowered expression of inflammatory response-related genes (e.g. *IL1B, IL17, IL22, IL36A, IL36G, IL36RN*) only at the end of treatment (week 2–3) (Table 3). Notably, genes involved in T-cell activation were not differentially expressed in the observation period (during dithranol up to week 2–3). To verify a subset of differentially expressed genes from the microarray data, we performed nCounter Nanostring analysis on 80 target and four reference genes. Ratios of microarray target genes strongly correlated with those obtained from Nanostring analysis at the early (day 6) (r = 0.8830) and late time point examined at the end of dithranol treatment (week 2–3) (r = 0.8859) (Supplementary file 2). Comparing day 6 vs. baseline, we found gene ontology (GO) terms related to keratinization (e.g. keratinocyte differentiation, establishment of skin barrier) and neutrophil chemotaxis (e.g. neutrophil migration, regulation of neutrophil migration) among the most significant GO groups (Table 4). Top significantly enriched GO terms at week 2–3 vs. baseline were related to immune response (e.g. inflammatory response, cytokine secretion) and differentiation of keratinocytes (e.g. epidermis development, keratinization) (Table 5). At follow-up (4–6 weeks after end of treatment), 10 of 13 patients showed >50% reduction in epidermal thickness and >75% reduction in Ki67 staining. These 10 patients were classified as histological responders. Microarray gene expression analysis at end of treatment (week 2–3) revealed that 131 genes were differentially expressed in histological responders compared to non-responders, predicting histological outcome for the follow-up time point 4–6 weeks after the end of treatment. Using gene ontology enrichment analysis of these DEGs, we found pathways like keratinocyte differentiation, cornification and keratin filament formation among the top 20 GO terms (Supplementary file 3).

**Table 3.** Top 45 differentially regulated genes in dithranol-treated lesional skin at end of treatment compared to baseline from 15 patients with psoriasis (p<0.05, fold change >1.5).

Probe set ID	Gene symbol	Gene title	End of treatment vs. baseline (fold change)
16947045	AADAC	Arylacetamide deacetylase	3.07
16765005	KRT73	Keratin 73	2.31
16976868	BTC	Betacellulin	2.27
16924792	CLDN8	Claudin 8	2.11
17125092	CNTNAP3	Contactin associated protein-like 3	2.09
17097358	SLC46A2	Solute carrier family 46, member 2	2.07
16780133	SLITRK6	SLIT and NTRK-like family, member 6	2.00
16834436	RAMP2	Receptor (G protein-coupled) activity modifying protein 2	1.96
17123970	ZDHHC11B	Zinc finger, DHHC-type containing 11B	1.88
17104313	AR	Androgen receptor	1.85
16951140	MIR548I2	MicroRNA 548i-2	1.85
17063366	ATP6V0A4	ATPase, H+ transporting, lysosomal V0 subunit a4	1.84
16974224	MIR548I2	MicroRNA 548i-2	1.83
16687914	CYP2J2	Cytochrome P450, family 2, subfamily J, polypeptide 2	1.82
17125106	CNTNAP3B	Contactin associated protein-like 3B	1.80
16903552	NEB	Nebulin	1.80
17125094	CNTNAP3B	Contactin associated protein-like 3B	1.80
16770284	TMEM116	Transmembrane protein 116	1.79
16765041	KRT77	Keratin 77	1.79
17123972	ZDHHC11B	Zinc finger, DHHC-type containing 11B	1.78
16688210	MIR3671	MicroRNA 3671	1.78
17125218	CNTNAP3	Contactin associated protein-like 3	1.74
16764923	KRT6A	Keratin 6A	−3.69
16767261	IL22	Interleukin 22	−3.87
17065453	DEFB103A	Defensin, beta 103A	−3.94
17074366	DEFB103A	Defensin, beta 103A	−3.94
16671027	LCE3C	Late cornified envelope 3C	−4.14
16743751	MMP12	Matrix metallopeptidase 12 (macrophage elastase)	−4.42
16979444	TNIP3	TNFAIP3 interacting protein 3	−4.48
17106398	SLC6A14	Solute carrier family 6 (amino acid transporter), member 14	−4.60
16686734	CYP4Z2P	Cytochrome P450, family 4, subfamily Z, polypeptide 2	−4.89
16671144	S100A7A	S100 calcium binding protein A7A	−5.00
16764907	KRT6C	Keratin 6C	−5.01
16730157	HEPHL1	Hephaestin-like 1	−5.15
16967831	EPGN	Epithelial mitogen	−5.31
16842517	NOS2	Nitric oxide synthase 2, inducible	−5.38
16693409	S100A12	S100 calcium binding protein A12	−6.12
16813112	RHCG	Rh family, C glycoprotein	−6.18
16738803	TCN1	Transcobalamin I (vitamin B12 binding protein, R binder family)	−6.45
16924785	CLDN17	Claudin 17	−8.09
16693365	SPRR2C	Small proline-rich protein 2C (pseudogene)	−8.72
16967771	CXCL8	Chemokine (C-X-C motif) ligand 8	−9.14
17074361	DEFB4B	Defensin, beta 4B	−9.39
16693375	SPRR2F	Small proline-rich protein 2F	−10.12
16884602	IL36A	Interleukin 36, alpha	−10.50

**Table 4.** Top 20 significantly enriched pathways as determined by Gene Ontology (GO) enrichment analysis in dithranol-treated lesional skin after 6 days compared to baseline from 15 patients with psoriasis. (GO was done using Cytoscape software [Bindea et al., 2009; Shannon et al., 2003]; a.o. = among others).

GOID	GO term	P-Value	% Associated Genes	Associated genes found
GO:0001533	Cornified envelope	3.8E-12	10.45	FLG, HRNR, KRT2, LCE1A, LCE1C, LCE2D, SPRR2F
GO:0031424	Keratinization	7.7E-12	3.93	CASP14, FLG, HRNR, KRT2, KRT73, LCE1A, LCE1C, LCE2D, SPRR2F
GO:0030216	Keratinocyte differentiation	79.0E-12	3.02	CASP14, FLG, HRNR, KRT2, KRT73, LCE1A, LCE1C, LCE2D, SPRR2F
GO:0018149	Peptide cross-linking	300.0E-12	9.84	FLG, KRT2, LCE1A, LCE1C, LCE2D, SPRR2F
GO:0070268	Cornification	12.0E-9	5.31	CASP14, FLG, KRT2, KRT73, LCE1A, SPRR2F
GO:0004168	Receptor CXCR2 binds ligands CXCL1 to 7	1.0E-6	33.33	CXCL5, CXCL8, PPBP
GO:0042379	Chemokine receptor binding	5.4E-6	6.25	CXCL5, CXCL8, DEFB4A, PPBP
GO:0045236	CXCR chemokine receptor binding	6.0E-6	18.75	CXCL5, CXCL8, PPBP
GO:0061436	Establishment of skin barrier	18.0E-6	13.04	FLG, FLG2, HRNR
GO:0033561	Regulation of water loss via skin	21.0E-6	12.00	FLG, FLG2, HRNR
GO:0004657	IL-17 signaling pathway	21.0E-6	4.30	CXCL5, CXCL8, DEFB4A, DEFB4B
GO:0007874	Keratin filament formation	21.0E-6	4.17	FLG, KRT2, KRT73, SPRR2F
GO:0030593	Neutrophil chemotaxis	21.0E-6	4.17	CXCL5, CXCL8, PPBP, TREM1
GO:1990266	Neutrophil migration	27.0E-6	3.85	CXCL5, CXCL8, PPBP, TREM1
GO:0071621	Granulocyte chemotaxis	38.0E-6	3.31	CXCL5, CXCL8, PPBP, TREM1
GO:1902622	Regulation of neutrophil migration	45.0E-6	7.50	CXCL5, CXCL8, PPBP
GO:0071622	Regulation of granulocyte chemotaxis	71.0E-6	5.66	CXCL5, CXCL8, PPBP
GO:0030104	Water homeostasis	130.0E-6	4.00	FLG, FLG2, HRNR
GO:0002690	Positive regulation of leukocyte chemotaxis	120.0E-6	3.30	CXCL5, CXCL8, PPBP
GO:0004867	Serine-type endopeptidase inhibitor activity	79.0E-6	3.00	SERPINB1, SERPINB12, SPINK7

**Table 5.** Top 20 significantly enriched pathways as determined by Gene Ontology (GO) enrichment analysis in dithranol-treated lesional skin at end of treatment compared to baseline from 15 patients with psoriasis. (GO was done using Cytoscape software; Bindea et al., 2009; Shannon et al., 2003 a.o. = among others).

GOID	GO term	P-Value	% Associated Genes	Associated genes found
GO:0006954	Inflammatory response	17.0E-18	6.93	IL17A, IL1B, IL20, IL22, IL36A, IL36G, IL36RN, a.o.
GO:0006952	Defense response	52.0E-18	4.60	CXCL8, CXCL9, DEFB103A, DEFB4A, IFNG, IL17A, IL1B, IL20, IL22, IL36A, IL36G, IL36RN, IL4R, IRAK2, IRF1, KRT16, a.o.
GO:0051707	Response to other organism	56.0E-18	6.02	CCL2, CCL20, CCL22, CD24, CD80, COTL1, CXCL13, CXCL16, CXCL8, CXCL9, DDX21, DEFB103A, DEFB4A, a.o.
GO:0050663	Cytokine secretion	480.0E-18	13.78	IFNG, IL17A, IL1B, IL26, IL36RN, IL4R, LYN, MMP12, NOS2, PAEP, PANX1, PNP, S100A12, S100A8, S100A9, a.o.
GO:0001816	Cytokine production	680.0E-18	6.76	IDO1, IFNG, IL17A, IL1B, IL26, IL36A, IL36RN, IL4R, IRF1, LTF, LYN, MB21D1, MMP12, NOS2, a.o.
GO:0012501	Programmed cell death	1.0E-15	4.05	CASP5, CASP7, CCL2, CCL21, CD24, CD274, CD38, IFNG, IL17A, IL1B, IRF1, IVL, KLK13, KRT16, KRT17, KRT31, KRT6A, KRT6C, KRT73, KRT74, KRT77, a.o.
GO:0051240	Positive regulation of multicellular organismal process	1.5E-15	4.57	CXCL17, CXCL8, FBN2, FERMT1, GBP5, GPR68, HPSE, HRH2, IDO1, IFNG, IL17A, IL1B, IL20, IL26, IL36A, S100A8, S100A9, SERPINB3, SERPINB7, a.o.
GO:0001817	Regulation of cytokine production	2.0E-15	7.04	CCL2, CCL20, CD24, CD274, CD80, CD83, CXCL17, IFNG, IL17A, IL1B, IL26, IL36A, IL36RN, IL4R, IRF1, TNFRSF9, WNT5A, a.o.
GO:0002237	Response to molecule of bacterial origin	15.0E-15	9.22	S100A8, S100A9, SELE, SOD2, TIMP4, TNFRSF9, TNIP3, WNT5A, ZC3H12A, a.o.
GO:0070268	Cornification	100.0E-15	17.70	DSC2, DSG3, IVL, KLK13, KRT16, KRT17, KRT31, KRT6A, KRT6C, KRT73, KRT74, KRT77, PI3, SPRR2A, SPRR2B, SPRR2D, a.o.
GO:0043588	Skin development	190.0E-15	8.17	IL20, IVL, KLK13, KRT16, KRT17, KRT31, KRT6A, KRT6C, KRT73, KRT74, KRT77, LCE3A, LCE3C, LCE3E, a.o.
GO:0008544	Epidermis development	220.0E-15	7.63	DSC2, DSG3, EPHA2, FERMT1, FOXE1, FURIN, HPSE, IL20, IVL, KLK13, KRT16, KRT17, KRT31, KRT6A, KRT6C, KRT73, KRT74, KRT77, a.o.
GO:0009617	Response to bacterium	240.0E-15	6.63	CCL2, CCL20, CD24, CD80, CXCL13, CXCL16, CXCL8, CXCL9, DEFB103A, DEFB4A, S100A12, S100A8, S100A9, TREM1, a.o.
GO:0001819	Positive regulation of cytokine production	1.4E-12	7.89	CCL2, CCL20, CD274, CD80, CD83, CXCL17, FERMT1, GBP5, HPSE, IDO1, IFNG, IL17A, IL1B, IL26, IL36A, a.o.
GO:0050707	Regulation of cytokine secretion	2.4E-12	13.10	AIM2, CASP5, CD274, FERMT1, GBP1, IFNG, IL17A, IL1B, IL26, IL36RN, IL4R, LYN, MMP12, PAEP, PANX1, a.o.
GO:0005125	Cytokine activity	4.4E-12	10.64	CCL20, CCL21, CCL22, CCL4L2, CXCL13, CXCL16, CXCL8, CXCL9, FAM3D, IFNG, IL17A, IL1B, IL20, IL22, IL26, a.o.
GO:0031424	Keratinization	21.0E-12	10.48	IVL, KLK13, KRT16, KRT17, KRT31, KRT6A, KRT6C, KRT73, KRT74, KRT77, LCE3A, LCE3C, LCE3E, PI3, SPRR2A, SPRR2B, a.o.
GO:0002790	Peptide secretion	97.0E-12	6.25	CD274, CD38, DOC2B, FAM3D, FERMT1, GBP1, GBP5, GLUL, GPR68, IFNG, IL17A, IL1B, IL26, IL36RN, IL4R, LYN, MMP12, NOS2, TREM1, WNT5A, a.o.
GO:0032940	Secretion by cell	140.0E-12	4.04	AIM2, AMPD3, CASP5, IL36RN, IL4R, LCN2, LRG1, LTF, LYN, MMP12, NOS2, NR1D1, NR4A3, OLR1, PAEP, PANX1, PLA2G3, a.o.
GO:0009913	Epidermal cell differentiation	190.0E-12	7.91	DSC2, DSG3, EPHA2, FURIN, IL20, IVL, KLK13, KRT16, KRT17, KRT31, KRT6A, KRT6C, KRT73, LCE3E, PI3, SLITRK6, SPRR2A, SPRR2B, SPRR2D, SPRR2E, a.o.

### Topical application of dithranol ameliorates psoriasis-like skin lesions in c-Jun/JunB knockout mice by directly targeting keratinocyte genes and inhibiting *IL36RN*

**Figure 3.**
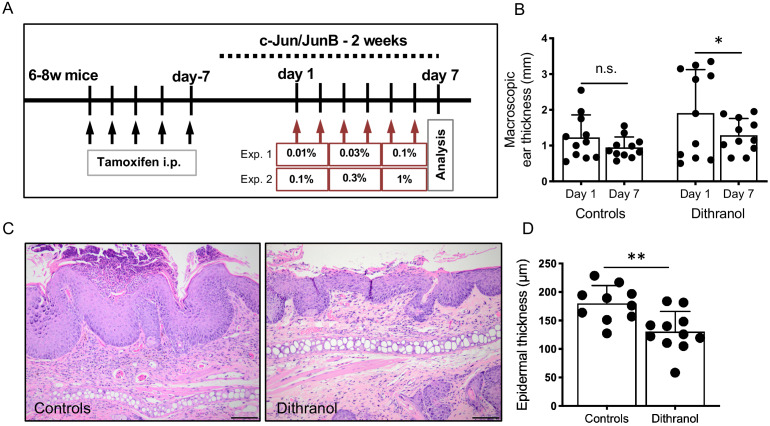
Topical application of dithranol ameliorates psoriasis-like skin lesions in c-Jun/JunB knockout mice. (**A**) Schematic representation of experimental set-up. Red arrows indicate dithranol application in series of increasing concentrations. (Exp. 1 and 2 = experiment 1 and 2) (**B**) Macroscopic ear thickness on day 1 compared to day 7. Dithranol treatment led to a significant reduction in ear thickness. Controls (n = 11), dithranol group (n = 11); Paired t-test was used for statistics. (**C**) Representative H and E images of untreated and dithranol-treated ears. (**D**) Dithranol treatment led to a significant reduction in epidermis thickness. Controls (n = 10), dithranol group (n = 11); unpaired t-test was used for statistics. Data from the two experiments was pooled (**D**). Bars represent mean ± SD; n.s. = not significant; *p≤0.05; **p≤0.01; ***p≤0.001; ****p≤0.0001; scale bar = 100 µm. Figure 3—source data 1.Values displayed in scatter plots shown in Figure 3.

**Figure 4.**
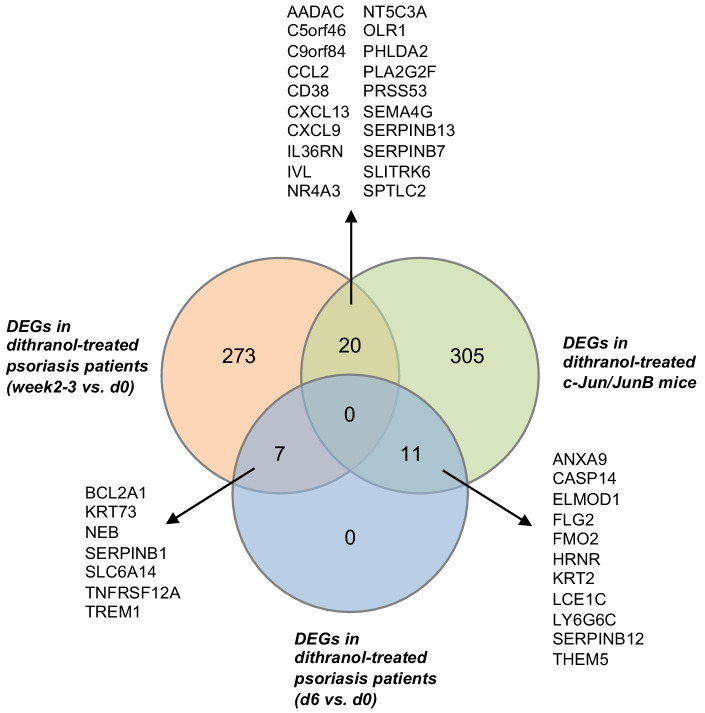
Venn diagram (created using InteractiVenn Heberle et al., 2015) showing comparison and overlap between differentially expressed genes (DEGs) in dithranol-treated human psoriatic skin at week 2–3 vs. day 0, DEGs in dithranol-treated human psoriatic skin at day 6 vs. day 0 and DEGs in dithranol-treated c-Jun/JunB psoriatic skin.

To study the effect of dithranol in a keratinocyte-based psoriatic mouse model, we used c-Jun/JunB knockout mice (treatment protocol shown in Figure 3A). Topical dithranol application strongly reduced psoriatic lesions as measured by macroscopic overall ear thickness and microscopic epidermal thickness in this genetic model of psoriasis based on inducible epidermal deletion of the AP1 transcription factors c-Jun/JunB (Glitzner et al., 2014; Zenz et al., 2005; Figure 3B–D). There was no difference in the outcome to dithranol with regard to different concentrations series, therefore, data was pooled for certain analyses as indicated (Figure 3). Gene expression profiling using Clariom S mouse microarray showed that among the top 45 differentially regulated genes, dithranol downregulated genes belonging to the group of late cornified envelope genes (*Lce6a, Lce1i, Lce1g, Lce1f*), as well as hornerin (*Hrnr*) (Table 6). Gene ontology enrichment analysis of all differentially expressed genes (fold change >1.5 and p-value<0.05) revealed skin development, epidermis development, epithelial cell differentiation and keratinization among the top 20 significantly enriched pathways (Table 7). Associated genes found were for example *Flg2, Ivl, Hrnr, Lor, Krt2, Casp14, Lce1c, Serpinb7* and *Serpbinb13*, among others. We then compared DEGs from psoriasis patients treated with dithranol to DEGs from dithranol-treated c-Jun/JunB knockout mice (Figure 4). In total, the microarray assay allowed us to compare 300 DEGs in dithranol-treated psoriasis patients (week 2–3 vs. day 0) and 18 DEGs from day 6 vs. day 0 with 336 murine DEGs from dithranol-treated c-Jun/JunB knockout mice. Notably, comparing DEGs from day 6 vs. day 0 with murine DEGs, we found an overlap of 11 genes. Among these genes were *CASP14*, a non-apoptotic caspase involved in epidermal differentiation and *FLG2, HRNR, KRT2, LCE1C* and *SERPINB12*, involved in keratinocyte differentiation and epidermis development (Bergboer et al., 2011; Henry et al., 2011; Henry, 2012; Kuechle et al., 2001; Sandilands et al., 2009; Sivaprasad et al., 2015; Toulza et al., 2007). Even more strikingly, the overlap between DEGs from week 2–3 vs. day 0 and murine DEGs comprised 20 genes including *IL36RN*, *IVL, SERPINB7* and *SERPINB13*, that were all increased in lesional skin at baseline compared to non-lesional skin and downregulated by dithranol treatment. Findings from microarray analysis were verified by RT-PCR (Figure 4—figure supplement 1). The group of genes encoding serpins, as well as involucrin have been shown to play a role in keratinocyte differentiation (Henry, 2012; Toulza et al., 2007) and have been associated with psoriasis (Roberson and Bowcock, 2010; Suárez-Fariñas et al., 2012; Wolf et al., 2012), belonging to the ‘psoriasis transcriptome’ identified by Tian et al., 2012. In addition, dithranol downregulated expression of elevated *IL36A* and *IL36G* in human psoriatic skin and *IL36B (Il1f8)* in c-Jun/JunB psoriatic skin. To substantiate dithranol’s effect on keratinocytes, we employed the mouse-tail test, a traditional model to quantify the effect of topical anti-psoriatics on keratinocyte differentiation by measuring degree of orthokeratosis versus parakeratosis (Bosman et al., 1992; Sebök et al., 2000; Wu et al., 2015). We found a strong increase in percentage of orthokeratosis (from 18.8 to 63.4%) reflecting dithranol’s keratinocyte differentiation-inducing activity (Figure 3—figure supplement 1), consistent with previous work (Bosman et al., 1992; Hofbauer et al., 1988; Sebök et al., 2000; Wrench and Britten, 1975). Next, we performed RT-PCRs of a selected panel of keratinocyte differentiation markers, AMPs and inflammatory markers (based on our microarray data) of dithranol-treated murine tail skin. We found a strong upregulation of keratinization markers (*Flg, Krt16, Serpinb3a*) and several AMPs (*Lcn2, S100a8, S100a9, Defb3)*) (Figure 3—figure supplement 2). Interestingly, dithranol downregulated expression of the antimicrobial peptide *Camp/LL37*, as well as *Cxcl5*, a chemotactic factor for neutrophils.

**Figure 3—figure supplement 1.**
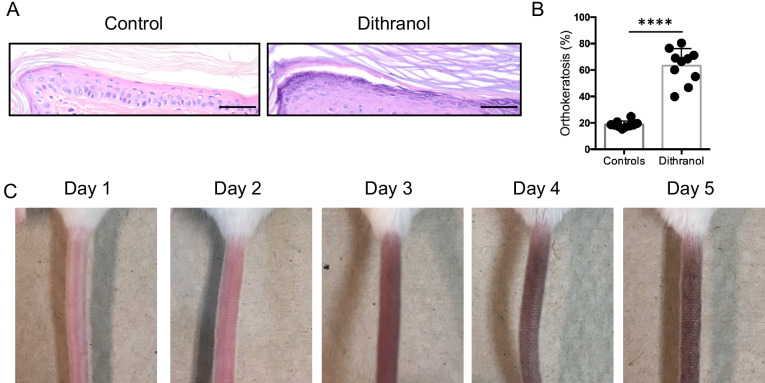
Strong inducing effect of dithranol in the mouse-tail test. (**A**) Representative H and E images of vehicle-treated and dithranol-treated mouse tail skin. (**B**) Dithranol treatment significantly increased orthokeratosis. (**C**) Representative images of erythema and typical brownish discoloration of skin caused by dithranol. Bars represent mean ± SD (n = 10). Mann-Whitney test was used for statistics. *p≤0.05; **p≤0.01; ***p≤0.001; ****p≤0.0001; Scale bar = 50 µm.

**Figure 3—figure supplement 2.**
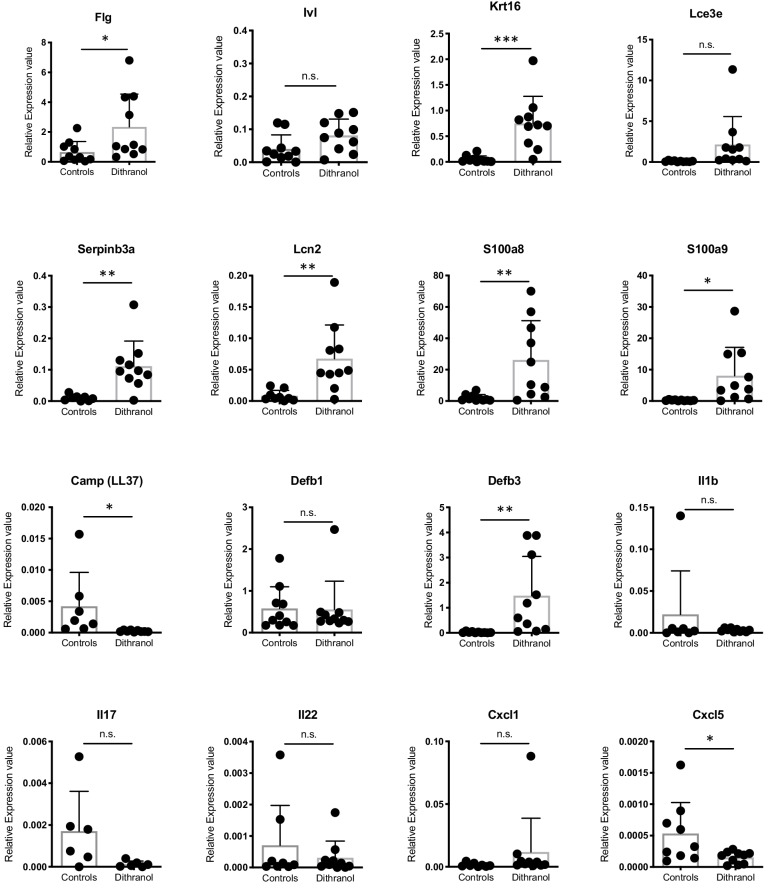
Gene expression analysis by RT-PCR of keratinization markers (Flg, Ivl, Krt16, Lce3e, Serpinb3a), AMPs (Lcn2, S100a8, S100a9, Camp, Defb1, Defb3) and inflammatory markers (Il1b, Il17, Il22, Cxcl1, Cxcl5) of dithranol- and vehicle-treated murine tail skin. Bars represent mean ± SD (n = 10) of relative expression values (deltaCT) normalized to Ubc. Unpaired t-test was used for statistics (n.s. = not significant) Flg, filaggrin; Ivl, involucrin, Krt16, keratin16; Lce3e, Late Cornified Envelope 3e; Camp, Cathelicidin Antimicrobial Peptide; Defb1, Beta-Defensin 1; Defb3, Beta-Defensin 3; Il1b, Interleukin one beta; Il17, Interleukin 17; Il22, Interleukin 22; Cxcl1, C-X-C Motif Chemokine Ligand 1; Cxcl5, C-X-C Motif Chemokine Ligand 5.

**Figure 3—figure supplement 3.**
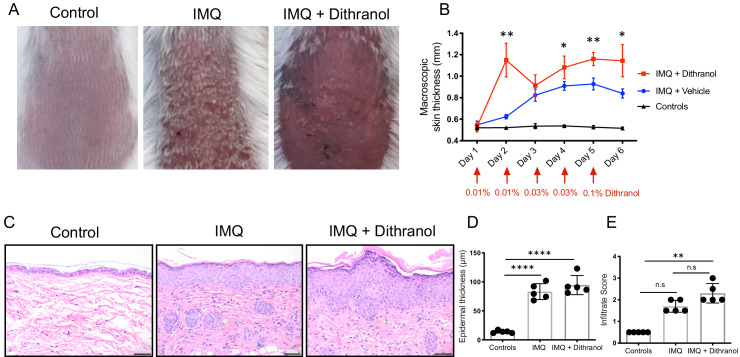
Dithranol causes exacerbation of psoriasis-like skin lesions in imiquimod mouse model. (**A**) Representative images of untreated, IMQ-treated and IMQ and dithranol-treated dorsal skin of mice. (**B**) Macroscopic skin thickness (red arrows indicate concentration of dithranol), (**C–D**) epidermal thickness measurements with corresponding H and E images and E) cellular infiltrate score show exacerbation of psoriasis-like dermatitis in IMQ and Dithranol-treated mice. Infiltrate score (0 = none, 0.5 = none/low, 1 = low, 1.5 = low/moderate, 2 = moderate, 2.5 = moderate/high, 3 = high infiltration of immune cells); Tukey’s multiple comparisons test (**B**), One-way ANOVA with Tukey’s multiple comparisons test (**D**) and Kruskal Wallis test with Dunn’s multiple comparisons test (**E**) was used for statistics. Bars represent mean ± SD (n = 5); *p≤0.05; **p≤0.01; ***p≤0.001; ****p≤0.0001; Scale bar = 50 µm.

**Figure 4—figure supplement 1.**
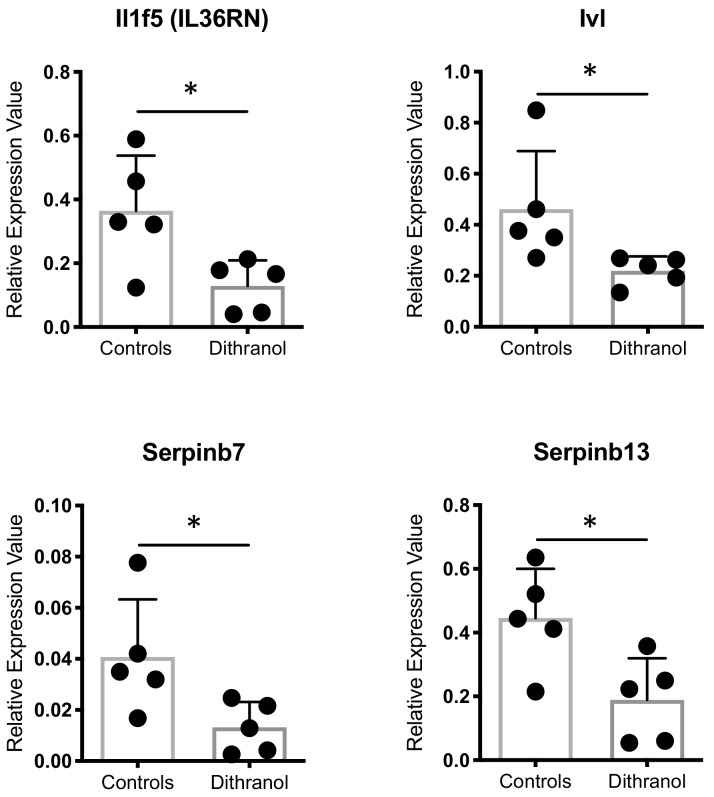
Verification of microarray gene expression analysis: Gene expression analysis by RT-PCR of selected genes based on microarray data in dithranol-treated c-Jun/JunB knockout mice vs. vehicle-treated controls. Bars represent mean ± SD (n = 5) of relative expression values (deltaCT) normalized to Ywhaz. Unpaired t-test was used for statistics. Il1f5 (IL36RN), interleukin 1 family member 5 (IL-36 receptor antagonist); Ivl, Involucrin; Serpinb7, Serine peptidase inhibitor, clade B member 7; Serpinb13, Serine peptidase inhibitor, clade B member 13.

**Table 6.** Top 45 differentially regulated genes in dithranol-treated psoriatic skin of c-Jun/JunB knockout mice compared to that of vehicle-treated controls (p<0.05, fold change (FC) >1.5).

Probe set ID	Gene symbol	Gene title	Fold change
TC0Y00000006.mm.2	Eif2s3y	Eukaryotic translation initiation factor 2, subunit 3, structural gene Y-linked	10,37
TC0Y00000233.mm.2	Uty	Ubiquitously transcribed tetratricopeptide repeat gene, Y chromosome	6,38
TC0Y00000235.mm.2	Ddx3y	DEAD (Asp-Glu-Ala-Asp) box polypeptide 3, Y-linked	5,40
TC0900000047.mm.2	Mmp3	Matrix metallopeptidase 3	3,64
TC0Y00000079.mm.2	Ssty2	Spermiogenesis specific transcript on the Y 2	3,31
TC0100001632.mm.2	Ifi209	Interferon activated gene 209	3,23
TC0500002755.mm.2	Cxcl9	Chemokine (C-X-C motif) ligand 9	2,85
TC0300003133.mm.2	Ifi44	Interferon-induced protein 44	2,69
TC0100003591.mm.2	Ifi213	Interferon activated gene 213	2,53
TC0100001634.mm.2	Ifi208	Interferon activated gene 208	2,49
TC1900000217.mm.2	Ms4a4c	Membrane-spanning 4-domains, subfamily A, member 4C	2,45
TC0300002684.mm.2	Chil3	Chitinase-like 3	2,39
TC0100003550.mm.2	Slamf7	SLAM family member 7	2,33
TC0500000922.mm.2	Cxcl13	Chemokine (C-X-C motif) ligand 13	2,26
TC1900000500.mm.2	Ifit2	Interferon-induced protein with tetratricopeptide repeats 2	2,20
TC0500002840.mm.2	Plac8	Placenta-specific 8	2,20
TC0700001630.mm.2	Adm	Adrenomedullin	2,15
TC1900000501.mm.2	Ifit3	Interferon-induced protein with tetratricopeptide repeats 3	2,13
TC1800000610.mm.2	Iigp1	Interferon inducible GTPase 1	2,07
TC0300001446.mm.2	Gbp3	Guanylate binding protein 3	2,07
TC0300003134.mm.2	Ifi44l	Interferon-induced protein 44 like	2,05
TC1900000502.mm.2	Ifit3b	Interferon-induced protein with tetratricopeptide repeats 3B	2,02
TC0400003296.mm.2	Skint5	Selection and upkeep of intraepithelial T cells 5	−2,37
TC0300000482.mm.2	Aadac	Arylacetamide deacetylase	−2,37
TC1100000469.mm.2	Fndc9	Fibronectin type III domain containing 9	−2,39
TC0300002399.mm.2	Lce6a	Late cornified envelope 6A	−2,42
TC0300002409.mm.2	Lce1i	Late cornified envelope 1I	−2,44
TC0300002412.mm.2	Kprp	Keratinocyte expressed, proline-rich	−2,44
TC0300000846.mm.2	Hrnr	Hornerin	−2,48
TC0300002407.mm.2	Lce1g	Late cornified envelope 1G	−2,49
TC1600000324.mm.2	Fetub	Fetuin beta	−2,53
TC1300001377.mm.2	Akr1c18	Aldo-keto reductase family 1, member C18	−2,56
TC0300002406.mm.2	Lce1f	Late cornified envelope 1F	−2,57
TC1500001714.mm.2	Slurp1	Secreted Ly6/Plaur domain containing 1	−2,57
TC1500002344.mm.2	Gsdmc2	Gasdermin C2	−2,59
TC1000000145.mm.2	Il20ra	Interleukin 20 receptor, alpha	−2,66
TC0700000443.mm.2	Cyp2b19	Cytochrome P450, family 2, subfamily b, polypeptide 19	−2,70
TC0100000103.mm.2	Ly96	Lymphocyte antigen 96	−2,89
TC0700000778.mm.2	Klk5	Kallikrein related-peptidase 5	−3,15
TC0300003252.mm.2	Clca3a2	Chloride channel accessory 3A2	−3,20
TC0900002312.mm.2	Elmod1	ELMO/CED-12 domain containing 1	−3,20
TC0300002401.mm.2	Lce1a1	Late cornified envelope 1A1	−3,26
TC0700000484.mm.2	Fcgbp	Fc fragment of IgG binding protein	−3,83
TC0300002405.mm.2	Lce1e	Late cornified envelope 1E	−4,16
TC1500002274.mm.2	Krt2	Keratin 2	−5,95

**Table 7.** Top 20 significantly enriched pathways as determined by Gene Ontology (GO) enrichment analysis in dithranol-treated psoriatic skin of c-Jun/JunB knockout mice compared to that of vehicle-treated controls. (GO was done using Cytoscape software Bindea et al., 2009; Shannon et al., 2003; a.o. = among others).

Go id	GO term	P-Value	% Associated Genes	Associated genes found
GO:0043588	Skin development	14,0E-18	11,11	Casp14, Cldn1, Flg2, Gjb3, Hrnr, Ivl, Krt2, Lce1a1, Lce1a2, Lce1l, Lce1m, Lor, Pou2f3, Ptgs1, Scel, Tfap2b, a.o.
GO:0008544	Epidermis development	73,0E-15	9,18	Acer1, Alox8, Casp14, Cnfn, Cst6, Dnase1l2, Hrnr, Ivl, Krt2, Lce1c, Lce1d, Lce1e, Lce1f, Lce1g, Lce1h, Lce1i, Lce1j, a.o.
GO:0071345	Cellular response to cytokine stimulus	1,7E-12	6,26	Ccdc3, Ccl2, Ccl5, Ccr9, Cxcl13, Cxcl9, Edn1, Il18, Il1f5, Il1f8, Il1rl1, Il20ra, Stat2, a.o.
GO:0034097	Response to cytokine	7,1E-12	5,62	Cd38, Chad, Cldn1, Csf3, Edn1, Gbp2, Gbp3, Gbp4, Gbp7, Gbp8, Gbp9, Gm4951, Ifi203, Ifi204, Ifi209, Ifit1, Ifit2, Ifit3, Ifit3b, Xaf1, a.o.
GO:0035456	Response to interferon-beta	12,0E-12	25,00	Gbp2, Gbp3, Gbp4, Gm4951, Ifi203, Ifi204, Ifi209, Ifit1, Ifit3, Iigp1, Irgm1, Xaf1
GO:0006952	Defense response	63,0E-12	4,11	Ccl2, Ccl5, Cd180, Cd59a, Cxcl13, Cxcl9, Cybb, Defb6, Drd1, Herc6, Hp, Il18, Il1f5, Il1f8, Il1rl1, Irgm1, Kalrn, Klk5, a.o.
GO:0030855	Epithelial cell differentiation	430,0E-12	5,51	Casp14, Cdkn1a, Cldn1, Cnfn, Dlx3, Dnase1l2, Gsdmc2, Gstk1, Hrnr, Ivl, Klf15, Krt2, Lce1a1, Lor, Pou2f3, a.o.
GO:0020005	Symbiont-containing vacuole membrane	1,4E-9	66,67	Gbp2, Gbp3, Gbp4, Gbp7, Gbp9, Iigp1
GO:0044216	Other organism cell	5,6E-9	30,77	C4b, Gbp2, Gbp3, Gbp4, Gbp7, Gbp9, Iigp1, Tap1
GO:0044406	Adhesion of symbiont to host	120,0E-9	37,50	Ace2, Gbp2, Gbp3, Gbp4, Gbp7, Gbp9
GO:0045087	Innate immune response	390,0E-9	4,35	Ccl2, Ccl5, Cd180, Cfb, Cldn1, Ifit3, Iigp1, Il1f5, Il1f8, Irgm1, Lbp, Ly96, Mx1, Parp9, Sla, Slamf6, Slamf7, Stat2, Tlr7, Trim62, a.o.
GO:0034341	Response to interferon-gamma	1,0E-6	10,58	Ccl2, Ccl5, Cldn1, Edn1, Gbp2, Gbp3, Gbp4, Gbp7, Gbp8, Gbp9, Parp9
GO:0006954	Inflammatory response	5,9E-6	4,29	C3, C4b, Ccl2, Ccl5, Cd180, Cd59a, Chil3, Crip2, Ctla2a, Cxcl13, Cxcl9, Cybb, Hp, Il18, Il1f5, Il1f8, Il1rl1, Lbp, Ly96, a.o.
GO:0042832	Defense response to protozoan	9,4E-6	19,35	Gbp2, Gbp3, Gbp4, Gbp7, Gbp9, Iigp1
GO:0035457	Cellular response to interferon-alpha	21,0E-6	36,36	Ifit1, Ifit2, Ifit3, Ifit3b
GO:0030414	Peptidase inhibitor activity	22,0E-6	6,19	C3, C4b, Cst6, Ctla2b, Fetub, R3hdml, Serpinb12, Serpinb13, Serpinb2, Serpinb7, Spink14, Tfap2b, Wfdc12, Wfdc5
GO:0098542	Defense response to other organism	34,0E-6	4,05	Adm, Ccl5, Cxcl13, Cxcl9, Defb6, Gbp2, Gbp3, Hp, Ifit1, Ifit2, Ifit3, Ifit3b, Iigp1, Il1f5, Klk5, Lbp, Mx1, Oas1f, Plac8, Stat2, Tlr7, Wfdc12, a.o.
GO:0031424	Keratinization	43,0E-6	15,00	Casp14, Cnfn, Hrnr, Ivl, Krt2, Lor
GO:0044403	Symbiosis, encompassing mutualism through parasitism	55,0E-6	4,11	Ace2, Acta2, Atg16l2, Ccl5, Cxcl9, Gbp2, Gbp3, Gbp4, Gbp7, Gbp9, Ifit1, Ifit2, Ifit3, Ifit3b, Lbp, Mx1, Oas1f, Pou2f3, Rab9, Stat2, Tap1, Tlr7, Trim62

In contrast to the effects of dithranol in the c-Jun/JunB model and mouse-tail test, this agent had no therapeutic capacity in the immunologically mediated imiquimod (IMQ) mouse model, which is often referred to as a psoriatic-like skin inflammation model (van der Fits et al., 2009). Indeed, dithranol treatment worsened psoriatic lesions in that model. Overall skin thickness was significantly enhanced, consistent with an increase in epidermal thickness and worsened inflammation (as measured by cellular infiltrate score) in mice treated with both IMQ and dithranol compared to IMQ-treated mice (Figure 3—figure supplement 3). Different set-ups (i.e. simultaneous treatment with dithranol and IMQ and pre-treatment with IMQ for 5 days followed by dithranol treatment) were tested, but similar effects were observed (data not shown). Taken together, these results demonstrate that dithranol primarily targets keratinocytes and only has delayed effects on other immune cells such as T cells belonging to the IL-17/IL-23 axis in psoriatic skin.

## Discussion

This study demonstrates that topical dithranol directly targets keratinocytes (in particular their differentiation regulators and AMPs), keratinocyte-neutrophil crosstalk and inhibits the IL-36 inflammatory loop in psoriasis, thus unraveling after over 100 years of use, the therapeutic mechanism of one of the most effective topical treatments of psoriasis. Dithranol significantly diminished mRNA expression of pro-psoriatic IL-1 family members (*IL36A*, *IL36G* and *IL36RN*) (Supplementary file 1 and Figure 4). At day 6 after start of dithranol treatment, PASI had decreased by 33%, but at week 2–3 we saw a reduction of 58%, an effect that was paralleled by reduced expression of IL-36-related genes in psoriasis patients. The therapeutic importance of reduction of IL-1 family members in human skin was substantiated by results generated in the keratinocyte-based c-Jun/JunB mouse psoriasis model (Zenz et al., 2005). Expression of *IL36RN* (murine *Il1f5*) and *IL36B* (*Il1f8*) was significantly reduced in dithranol-treated psoriatic c-Jun/JunB lesions compared to controls (Figure 4) and among the top differentially expressed genes in our human and mouse dataset were genes involved in keratinocyte and epidermal differentiation (Table 4, Table 7). Dithranol strongly reduced mRNA expression of the keratinocyte differentiation regulator involucrin (*IVL*) and members of the serpin family (*SERPBINB7, SERBINB13*) both in lesional skin of patients and c-Jun/JunB knockout mice (Figure 4). Moreover, dithranol downregulated expression of AMPs such as ß-defensins (*DEFB4A* and *DEFB4B)* produced by keratinocytes (Liu et al., 2002; Schröder and Harder, 1999) within 6 days and chemotactic factors for neutrophils (such as *CXCL5* and *CXCL8*) (Albanesi et al., 2018; Table 2) and neutrophilic infiltration (as determined by MPO staining) within 2–3 weeks of treatment in human psoriatic skin (Figure 2).

Surprisingly, there were no significant changes in overall T cell numbers including CD4+ and CD8+ T cells, as well as FoxP3 positivity indicative for regulatory T cells in the skin in the early phase (within 6 days) during dithranol treatment. Reflecting dithranol’s primary effect on the epidermal compartment, the reduction of T cell numbers in the epidermis preceded that in the dermis. Whereas dithranol had decreased T cell counts in the epidermis at week 2–3, an effect on T cell numbers in the dermis was only evident at the follow-up visit, 4–6 weeks after the end of dithranol treatment (Figure 2), in agreement with previous reports favoring dithranol’s effect on keratinocytes (Holstein et al., 2017; Swinkels et al., 2002a; Vleuten et al., 1996; Yamamoto and Nishioka, 2003).

Vleuten et al., 1996 and Swinkels et al., 2002a applied immunohistochemistry to analyze differentiation and proliferation markers as well as T cell numbers in the skin and observed, as we did, a decrease in keratin 16, restoration of filaggrin and a decrease of Ki67 in the epidermis after 2 weeks of dithranol treatment. However, their data on the effect of dithranol on dermal T cell numbers at 4 weeks was controversial (Swinkels et al., 2002a; Vleuten et al., 1996). The group of Eberle recently tested the effects of dithranol using primary keratinocytes, a 3D psoriasis tissue model and some biopsy samples from psoriasis patients and reported on a reduction in Ki67 and keratin 16 positive cells in the epidermis using immunostaining and an inhibition of the antimicrobial peptide *DEFB4* using qPCR analysis. However, based on their observations, they concluded that dithranol’s anti-psoriatic effects cannot be explained by direct effects on keratinocyte differentiation or cytokine expression (Holstein et al., 2017).

Our genome-wide expression analysis indicates that dithranol primarily targets keratinocytes and that this is crucial for response to treatment, considering that differentially regulated genes in histological responders compared to non-responders belonged to pathways like keratinocyte differentiation, cornification and keratin filament formation (Supplementary file 3). The importance of dithranol’s direct effect on keratinocytes has been further substantiated by our findings generated using the mouse-tail model, a simple in vivo model to analyze effects of topical preparations on keratinocyte differentiation and parakeratosis (Bosman et al., 1992; Sebök et al., 2000; Wu et al., 2015). Similar to previous studies (Bosman et al., 1992; Hofbauer et al., 1988; Sebök et al., 2000; Wrench and Britten, 1975), we observed a strong increase in orthokeratosis after dithranol application in the mouse-tail test, reflecting its keratinocyte differentiation-inducing activity (Figure 3—figure supplement 1). Our transcriptional analysis indicated that the effect of dithranol in the mouse-tail test was linked to a strong upregulation of keratinocyte differentiation markers and several AMPs, while the pro-psoriatic antimicrobial peptide *Camp/LL37* was downregulated, as well as *Cxcl5*, a chemotactic factor for neutrophils (Figure 3—figure supplement 2), but not other pro-psoriatic AMPs (Fritz et al., 2017; Wang et al., 2018) such as *Defb3, S100a8* or *S100a9*. Although the mouse-tail test has evidently limitations since the disturbed cell differentiation of this model only reflects one of many aspects of psoriasis, it supports the primary effect of dithranol on keratinocytes with induced induction of orthokeratosis.

In contrast to its therapeutic effect in both keratinocyte-driven psoriasis models, the c-Jun/JunB model and mouse-tail test, dithranol did aggravate psoriatic lesions in the imiquimod model that has been solely shown to be immunologically mediated and dependent on the IL-17/IL-23 axis (van der Fits et al., 2009). In the latter model, biologics such as etanercept and anti-IL-17a agents (Liu et al., 2018), topical steroids (Sun et al., 2014) and vitamin d3 analogues (Germán et al., 2019) but also UVB and PUVA (Shirsath et al., 2018) were shown to have a beneficial effect. In contrast, dithranol significantly enhanced overall macroscopic skin thickness, consistent with a slight increase in epidermal hyperplasia and worsened inflammation (as measured by the density of cellular infiltrate in the dermis) (Figure 3—figure supplement 3). Dithranol treatment of healthy murine skin led to similar effects upon irritation, as it increased epidermal thickness and cellular infiltrate of the skin (data not shown). However, the irritant effect of dithranol may remain without functional anti-psoriatic relevance in human psoriasis (as indicated by the clinical results depicted in Figure 1—figure supplement 2 and discussed below) but might be crucial in the agent's therapeutic action in alopecia areata (Nasimi et al., 2019; Ngwanya et al., 2017).

**Figure 5.**
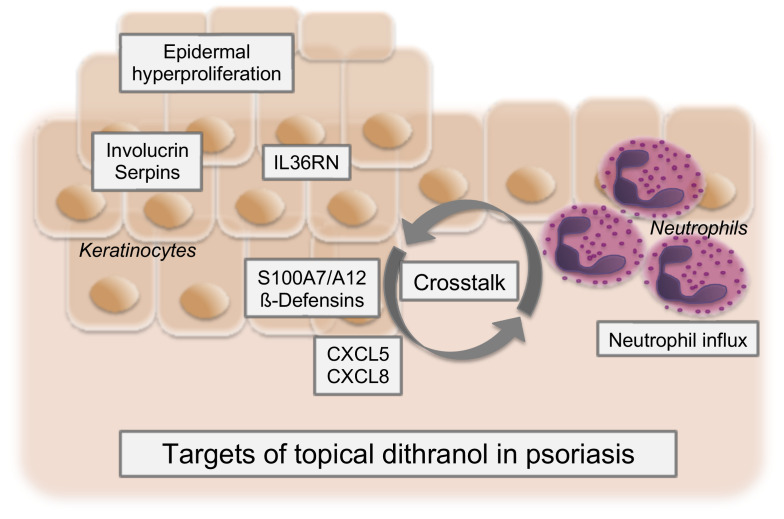
Proposed model of dithranol’s mechanism of action in psoriasis. Dithranol decreases expression of keratinocyte differentiation regulators (involucrin and serpins), IL-36-related genes, keratinocyte-derived antimicrobial peptides (AMPs) (S100A7/A12 and ß-defensins) and chemotactic factors for neutrophils (CXCL5, CXCL8). This disrupts the crosstalk between keratinocytes and neutrophils and leads to diminished neutrophilic infiltration, thereby halting the inflammatory feedback loop in psoriasis. Together, this results in clearance of psoriatic lesions.

Together, these data unambiguously demonstrate that dithranol directly acts on keratinocytes, their crosstalk with neutrophils and IL-36 signaling, with AMPs being the potential link (Figure 5). This goes also in line with the observation that Langerhans cells as another type of immune cells showed a delayed response to dithranol, with no changes during treatment and an evident increase in numbers only later on (at the follow-up visit 4–6 weeks after end of treatment). These results are consistent with previous studies performed by Swinkels et al., showing that there was no significant change in T cells or Langerhans cells during twelve days of dithranol treatment (Swinkels et al., 2002a).

Our findings on dithranol’s effect on members of the IL-1 family, being beneficial for its anti-psoriatic efficacy, are well in line with recent work on blockade of IL-36 pathway as a novel strategy for the treatment of pustular psoriasis (Bachelez et al., 2019) as well as plaque-type psoriasis (Benezeder and Wolf, 2019; Mahil et al., 2017). Notably, human keratinocytes express IL-1 family members (IL-36α, IL-36β, IL-36γ, IL-36Ra) and their receptor IL-36R (D'Erme et al., 2015; Foster et al., 2014; Johnston et al., 2017). Furthermore, normal human keratinocytes show increased expression of IL-1 group mRNA after treatment with psoriasis-associated cytokines (TNFα, IL-1α, IL-17,IL-22) (Johnston et al., 2011). Genome-wide association studies revealed that deficiency in interleukin-36 receptor antagonist due to IL36RN mutations was associated with generalized pustular psoriasis (GPP) (Marrakchi et al., 2011; Sugiura et al., 2013). Supporting this notion, blocking IL-36 receptor was effective in reducing epidermal hyperplasia and showed comparable effects to etanercept in a psoriatic skin xenotransplantation model (Blumberg et al., 2010). Furthermore, successful treatment of psoriasis with the anti-psoriatic standard treatment etanercept is accompanied by a decrease in *IL36A, IL36G* and *IL36RN* expression (Johnston et al., 2011). A recent clinical phase one study provided proof-of-concept for the efficacy of BI 655130, a monoclonal antibody against the interleukin-36 receptor in the treatment of generalized pustular psoriasis (Bachelez et al., 2019). Intriguingly, topical short-contact dithranol therapy is efficacious not only in plaque-type psoriasis, but under certain conditions (i.e. after stabilization of disease activity with bland emollients) reportedly also in pustular psoriasis (Farber and Nall, 1993; Gerritsen, 1999). In this neutrophilic-driven condition, in which one might expect that dithranol worsens a heavy inflammatory state, it may have beneficial capacity by targeting the IL-36 pathway and neutrophils, both playing a most crucial role in this type of psoriasis (Bachelez, 2018; Bachelez et al., 2019; Johnston et al., 2017; Marrakchi et al., 2011; Sugiura et al., 2013).

As outlined above, we observed a strong effect of dithranol on keratinocyte-neutrophil crosstalk. Importantly, early response to anti-IL-17a blockade, as one of the most effective biological treatments currently available, has been linked to inhibition of keratinocyte-neutrophil crosstalk (Reich et al., 2015). Similar to our observation, anti-IL-17a treatment with secukinumab significantly reduced epidermal hyperproliferation after 2 weeks, decreased mRNA expression of keratinocyte-derived chemotactic factors and led to a strong decrease in IL-17a positive neutrophils. Apparently, disrupting the crosstalk between keratinocytes and neutrophils may be a crucial early effect in the clinical efficacy of secukinumab in psoriasis (Reich et al., 2015). In addition, decreased expression of AMPs like ß-defensin and S100 proteins was observed after only 1 week of anti-IL-17a treatment with secukinumab (Krueger et al., 2019) and after 2 weeks of ixekizumab treatment (Krueger et al., 2012), well in line with our observed early effect of dithranol on AMPs. Krueger et al. concluded that clinical efficacy of anti-IL-17a treatment is closely linked to early inhibition of keratinocyte-derived products such as chemokines and AMPs. Together this suggests that dithranol’s direct action on keratinocytes at the molecular level may disrupt IL-17 pathway dysregulation (without directly blocking IL-17 or its receptor), leading in turn to similar downstream effects as treatment with anti-IL-17 antagonists.

Our study also negates the paradigm that dithranol-induced irritation is crucial for its anti-psoriatic action (Gerritsen, 1999; Kucharekova et al., 2005; Prins et al., 1998; van de Kerkhof, 1991; Wiegrebe and Müller, 1995). As depicted in Figure 1—figure supplement 2, there was no correlation between dithranol-induced perilesional as well as lesional erythema and its anti-psoriatic effect, indicating that dithranol’s irritation (Prins et al., 1998; Swinkels et al., 2002c; Swinkels et al., 2002b) is a treatment side-effect unrelated to its therapeutic mechanism. However, this irritant effect of dithranol may be crucial for its action in alopecia areata, a condition, in which it was shown to be greatly effective, leading to hair regrowth in a high percentage of cases (Ngwanya et al., 2017). Similar to other topical treatment options (Nasimi et al., 2019; Yoshimasu and Furukawa, 2016), an initial irritant reaction to dithranol is followed by regrowth of hair within weeks after treatment.

What our work does not answer, is how dithranol exactly acts at the molecular level. Cell culture studies have shown that dithranol targets mitochondria (McGill et al., 2005), alters cellular metabolism (Hollywood et al., 2015) and induces apoptosis in keratinocytes (George et al., 2013; McGill et al., 2005). Dithranol also inhibits human monocytes in vitro, inhibiting secretion of IL-6, IL-8 and TNFα (Mrowietz et al., 1992; Mrowietz et al., 1997). Its effects on neutrophils were also shown in vitro, where dithranol leads to an increase in superoxide generation and a decrease in leukotriene production in neutrophils (Kavanagh et al., 1996; Schröder, 1986). Moreover, the potential receptor of dithranol remains undefined. Anti-psoriatic effects of other topical treatment options have recently been linked to modulation of the aryl hydrocarbon receptor (AhR) (Smith et al., 2017) and AhR may play a role in pathogenesis of psoriasis (Di Meglio et al., 2014). However, it appears that dithranol does not act via modulation of AhR, as we did not observe differences in the response of skin to dithranol comparing AhR knockout mice to controls (data not shown). There is need for further studies investigating how dithranol exactly acts on the molecular level, which receptor it potentially binds to or inhibits or whether it acts through modulation of a specific transcription factor such as *NF-kB* or *STATs*. Another question is whether the effect of dithranol is specific compared to other topical treatments such as steroids and vitamin D3 analogues. There seem to be some overlapping mechanisms between dithranol and vitamin D3 analogues such as calcipotriol that has been shown to act on keratinocytes to repress the expression of IL-36α/γ, an effect mediated through keratinocytic vitamin D receptor (Germán et al., 2019). Moreover, similar to dithranol, calcipotriol decreased expression of AMPs such as ß-defensins in keratinocytes of psoriatic plaques (Peric et al., 2009). At the same time, calcipotriol normalized the proinflammatory cytokine milieu and decreased IL-17A, IL-17F and IL-8 transcript abundance in lesional psoriatic skin. Calcipotriol also directly targets Th17 cells (Fujiyama et al., 2016) and CD8+IL-17+ cells (Dyring-Andersen et al., 2015), whereas we found that dithranol only has delayed effects on T cells. Moreover, cathelicidin (LL37) expression was increased by calcipotriol (Peric et al., 2009), juxtaposing the results of dithranol treatment at least in the mouse-tail test of present study. That dithranol and vitamin D3 analogues may have similar basic mechanisms of action is also supported by the notion that depending on the concentration, both dithranol (Nasimi et al., 2019; Ngwanya et al., 2017) and calcipotriol (El Taieb et al., 2019; Fullerton et al., 1998; Molinelli et al., 2020) can be irritant to the skin and induce hair regrowth in alopecia areata. Compared to vitamin D3 analogues topical steroids have an even broader mechanisms of action in psoriasis linked to their anti-inflammatory, antiproliferative, vasoconstrictive (Uva et al., 2012) and immunomodulatory properties, in particular suppressing the IL-23/IL-17 axis, with IL-23 produced by dendritic cells/macrophages and IL-17 produced by Th17 cells/γδ T cells/innate lymphoid cells (Germán et al., 2019).

Another question that this study does not answer is, whether topical dithranol therapy has any effects on systemic psoriatic inflammation. However nowadays, treatment with dithranol is mainly administered in refractory, circumscribed psoriatic lesions in patients who do not have significant systemic inflammation how it may be otherwise the case in patients with moderate to severe forms of psoriasis.

Together our work opens up several avenues for novel topical (and potentially also systemic) treatment strategies in psoriasis. Not only targeting the IL-36 pathway, but also keratinocyte differentiation regulators (e.g. involucrin), keratinocyte-produced AMPs (ß-defensins like *DEFB4A, DEFB4B, DEFB103A*, S100 proteins like *S100A7, S100A12*), and neutrophils and their chemotactic factors (*CXCL5* and *CXCL8*) or members of the serpin family (*SERPINB7* and *SERPINB13*), are promising approaches. Such approaches may not only be helpful for chronic plaque-psoriasis, but also for pustular psoriasis, in which a vicious loop between AMPs such as cathelicidin (LL-37) and IL-36 signaling may drive psoriatic disease (Benezeder and Wolf, 2019; Furue et al., 2018; Li et al., 2014; Madonna et al., 2019; Ngo et al., 2018).

## Materials and methods

**Key resources table:** 

Reagent type (species) or resource	Designation	Source or reference	Identifiers	Additional information
Antibody	anti-CD1a; mouse monoclonal	Immunotech, Beckman Coulter	Clone: O10 RRID:AB_10547704	(undiluted)
Antibody	anti-CD3; mouse monoclonal	Novocastra, Leica Biosystems	Clone: PS1 RRID:AB_2847892	(1:100)
Antibody	anti-CD4; mouse monoclonal	Novocastra, Leica Biosystems	Clone: 1F6 RRID:AB_563559	(1:30)
Antibody	anti-CD8; mouse monoclonal	Dako, Agilent	Clone: C8/144b RRID:AB_2075537	(1:25)
Antibody	anti-CK16; rabbit monoclonal	Abcam	Clone: EPR13504 RRID:AB_2847885	(1:1000)
Antibody	anti-FoxP3; mouse monoclonal	Bio-Rad	Clone: 236A/E7 RRID:AB_2262813	(1:100)
Antibody	anti-Ki-67; mouse monoclonal	Dako, Agilent	Clone: MIB-1 RRID:AB_2631211	(1:50)
Antibody	anti-MPO; mouse monoclonal	Dako, Agilent	Clone: MPO-7 RRID:AB_578599	(1:100)
Strain, strain background *Mus musculus*	BALB/c, wild-type	Charles River Laboratories	RRID:IMSR_CRL:028 Charles River Strain code#: 028	
Strain, strain background *Mus musculus*	﻿JunB^f/f^ c-Jun^f/f^ K5-Cre-ER^T^	PMID:16163348		Obtained from the laboratory of Maria Sibilia (Medical University of Vienna)
Commercial assay or kit	miRNeasy Mini Kit	Qiagen	Cat #: 217004	
Commercial assay or kit	iScript Reverse Transcription Supermix	Bio-Rad	Cat #: 1708841	
Commercial assay or kit	GoTag qPCR Master Mix	Promega	Cat #: A6001	
Commercial assay or kit	Human GeneChip2.0 ST arrays	Affymetrix, ThermoFisher Scientific	Cat #:902113	
Commercial assay or kit	mouse Clariom S Assay	Affymetrix, ThermoFisher Scientific	Cat #:902919	
Commercial assay or kit	GeneChip WT PLUS Reagent Kit	ThermoFisher Scientific	Cat #: 902280	
Commercial assay or kit	GeneChip WT Terminal Labeling Kit	ThermoFisher Scientific	Cat #: 900671	
Commercial assay or kit	GeneChip Hybridization, Wash and Stain Kit	ThermoFisher Scientific	Cat #: 900720	
Commercial assay or kit	nCounter GX Custom codeset	NanoString Technologies		Custom codeset (80 target genes, four reference genes
Chemical compound, drug	Aldara (Imiquimod)5% cream	MEDA Pharmaceuticals	Cat #: 111981	
Chemical compound, drug	Tamoxifen	Sigma-Aldrich	Cat #: T5648	
Chemical compound, drug	Dithranol (1,8-Dihydroxy-9(10H)-anthracenone)	Gatt-Koller GmbH Pharmaceuticals	Cat #: 8069994	Dissolved in vaseline and provided by the pharmacy of the Medical University of Graz, Austria
Software, algorithm	GraphPad Prism version 8	GraphPad	RRID:SCR_002798 https://www.graphpad.com/scientific-software/prism/	
Software, algorithm	Interacti Venn	PMID:25994840	http://www.interactivenn.net/	
Software, algorithm	Cytoscape	PMID:19237447	RRID:SCR_003032 https://cytoscape.org/	
Software, algorithm	Transcriptome Analysis Console (TAC) 4.0.2	ThermoFisher Scientific	https://www.thermofisher.com/at/en/home/life-science/microarray-analysis/microarray-analysis-instruments-software-services/microarray-analysis-software/affymetrix-transcriptome-analysis-console-software.html	
Software, algorithm	Partek Genomics Suite version 6.6	Partek Inc	RRID:SCR_011860 https://www.partek.com/partek-genomics-suite/	
Software, algorithm	R Version 3.5.1	The R Project for Statistical Computing	RRID:SCR_001905 https://www.r-project.org/	
Software, algorithm	nSolver 2.5 Software	NanoString Technologies	https://www.nanostring.com/products/analysis-software/nsolver	

### Patients

#### Trial protocol and patient characteristics

For the clinical dithranol study, inclusion criteria were diagnosis of chronic plaque psoriasis, and age above 18 years. Exclusion criteria were intolerance of dithranol, autoimmune diseases, general poor health status, pregnancy and breast-feeding, topical treatment (steroids, vitamin D3-analogs and/or Vitamin A acid-derivates) within 2 weeks, and phototherapy within 4 weeks prior to study enrollment. None of the patients had received systemic treatment in the past prior to study enrollment. In total, 15 psoriasis patients (11 men, 4 women; median age 40.5 years, range 19.8–76.9 years) were enrolled. Mean duration of psoriasis had been 17.9 years (SD 11.5 years). Mean duration of dithranol treatment was 15.4 days (SD 3.6 days) and treatment was prematurely discontinued in two patients. Samples of normal skin from patients undergoing surgery for removal of benign skin lesions were available from 12 subjects (median age was 36.3 years, range 22.6–46.9 years) for control purposes. The samples were from lesion-adjacent, excised skin of patients who did not suffer from psoriasis, other inflammatory diseases or autoimmune diseases.

#### Patient treatment

Dithranol ointment was prepared in the hospital pharmacy with 2% salicylic acid and white vaseline as base. It was administered to the patients daily in increasing concentrations; dosage was adjusted individually to the level of skin irritation. Concentration was usually increased every other day (starting from 0.1%, next 0.16%, 0.2%, 0.4%, 1% and finally 2%) and mean treatment duration was 15.4 days.

#### Marker lesions and scoring

At each of the four visits (see Figure 1), i) Psoriasis Area and Severity index (PASI), ii) local psoriasis severity index (PSI) and erythema score of marker lesions and iii) perilesional erythema score were assessed. PSI was composed by rating of erythema, induration and scaling, each on a scale from 0 to 4, resulting in a maximum score of 12. Erythema score was determined by rating intensity of lesional erythema on a scale of 0–4 (0 = none, 1 = mild, 2 = moderate, 3 = severe, 4 = very severe erythema). For perilesional erythema score, intensity of perilesional erythema was rated on a scale of 0–3 (0 = none, 1 = mild, 2 = moderate, 3 = severe perilesional erythema). Per patient, four marker lesions of similar morphology and size and, if possible, from the same body regions or four marker areas (each 5 cm in diameter) of one or more larger psoriatic lesions were defined at study entry and then scored, treated with dithranol and later biopsy-sampled at certain timepoints.

#### Patient tissue sampling

Biopsy samples were taken from the psoriasis patients before (day 0), during early treatment at first strong perilesional inflammation between day 4 and 9 (with most biopsies taken at day 6) at end of treatment (approximately after 2–3 weeks) and at a follow-up visit (4–6 weeks after end of therapy). Per patient, a total of five biopsy samples were taken on four study days by means of a punch cylinder (up to 5 mm) under local anesthesia. At the first visit before starting therapy, one biopsy sample was taken additionally from adjacent non-lesional skin, with a distance of at least 5 cm from the edge of psoriatic skin. One part of each biopsy was fixed in 4% neutral-buffered paraformaldehyde and used for histology and immunohistochemistry. The other part was stored in RNAlater solution (Invitrogen, California, USA) at −80°C until RNA extraction for further analysis.

### Histology and immunohistochemistry

#### Analysis of HE stained sections

Human and murine samples fixed with paraformaldehyde were processed routinely, cut in 4 µm sections and stained with hematoxylin and eosin (HE). Five randomly selected fields per slide were investigated for histological analysis. Thickness of epidermis was measured from basal layer to stratum corneum using an Olympus BX41 microscope (Olympus Life Science Solutions, Hamburg, Germany), cellSens software (Olympus Life Science Solutions) and 20x magnification. Semi-quantitative scoring (0 = none, 0.5 = none/low, 1 = low, 1.5 = low/moderate, 2 = moderate, 2.5 = moderate/high, 3 = high density of infiltrate) was performed at five randomly selected locations per slide and at 20x magnification.

In the mouse-tail model, degree of orthokeratosis was analyzed as described by Bosman et al., 1992 . In brief, five randomly selected scales per sample were examined and the length of the granular layer (A) as well as the total length of the scale (B) were measured using cellSens software (Olympus-lifescience, Hamburg, Germany) and 20x magnification. The proportion of (A/B) x 100 depicts the percentage of orthokeratosis per scale.

#### Immunohistochemistry stainings and analysis

Antigen retrieval was performed using either EDTA-buffer (CD1a, CD3, CD4, CK16, CD8), citrate-buffer (FoxP3, Ki-67) or trypsin (MPO). Primary antibodies used were: anti-human CD1a (mouse monoclonal, clone O10, undiluted; Immunotech, Beckman Coulter, Praque, Czech Republic), anti-human CD3 (mouse monoclonal, clone PS1, dilution 1:100; Novocastra, Leica Biosystems, Mannheim, Germany), anti-human CD4 (mouse monoclonal, clone 1F6, dilution 1:30; Leica Biosystems), anti-human CD8 (mouse monoclonal, clone C8/144b, dilution 1:25, Dako Omnis, Agilent, Santa Clara, CA, USA), anti-human CK16 (rabbit monoclonal, clone EPR13504, dilution 1:1000, Abcam, Cambridge, UK), anti-human FoxP3 (clone 236A/E7, AbD Serotec, Bio-Rad, Hercules, CA, USA), anti-human Ki-67 (mouse monoclonal, clone MIB-1, dilution 1:50, Dako Omnis, Agilent) and anti-human MPO (mouse monoclonal, clone MPO-7, dilution 1:100, Dako Omnis, Agilent). Stainings were performed using the Dako REALTM Detection System, Peroxidase/AEC, rabbit/mouse (Dako, Agilent) on the Dako Autostainer Link 48 (Dako, Agilent) according to the manufacturer’s instructions. For quantification of CD1a, CD3, CD4, FoxP3, CD8, and MPO staining, all positively stained cells with visible nucleus in five randomly selected fields (separately for epidermis and dermis) per slide were counted and results were averaged to obtain mean cell counts. To quantify Ki-67 and CK16 staining, area of positive staining was divided by epidermal area as follows: one representative image per slide was taken on an Olympus BX41 microscope (Olympus Life Science Solutions, Hamburg, Germany) at 10x magnification using cellSens software (Olympus Life Science Solutions). TIFF images were imported into ImageJ program and pixels were converted to µm. Using the polygon sections tool, the outline of epidermis was framed, and the total area was measured. In addition, total area of particles (positively stained cells) within the epidermal area was assessed.

### Gene expression analyses

#### RNA extraction

Total RNA was extracted from frozen biopsies of psoriasis patients and control subjects. To facilitate homogenization, tissues were cut in 20 µm sections using a cryomicrotome and collected in precooled MagNA Lyser Green Beads tubes (Roche, Basel, Switzerland) and disruption of tissue was performed on the MagNA Lyser Instrument (Roche, Basel, Switzerland). After efficient homogenization, total RNA was extracted using the miRNeasy Mini Kit (Qiagen, Hilden, Germany), according to the manufacturer’s instructions. To ensure complete DNA removal, on-column DNase digestion was performed and RNA was eluted in 15–20 µl RNase-free water. Mouse tissues were handled in the same way except that sufficient homogenization of samples was obtained without using a cryomicrotome.

#### Microarray and pathway analysis

Isolated RNA was quality checked on a Bioanalyzer BA2100 (Agilent; Foster City, CA) using the RNA 6000 Nano LabChip according to manufacturer’s instructions and samples with RNA integrity numbers (RIN) between 5 to 8 were considered for analysis using Human GeneChip 2.0 ST arrays (Affymetrix, ThermoFisher Scientific, Waltham, MA, USA; Cat.No.: 902113) for the human samples and mouse Clariom S Assay (Affymetrix, ThermoFisher Scientific, Waltham, MA, USA; Cat No. 902919) for the mouse samples. For first and second strand cDNA synthesis 500 ng total RNA were used in the GeneChip WT PLUS Reagent Kit (ThermoFisher) according to manufacturer’s instructions. Fragmentation and terminal labelling was performed using the GeneChipTM WT Terminal Labeling Kit (ThermoFisher) and hybridization, wash and stain of arrays was performed on a GeneChip Fluidics 450 station using the GeneChip Hybridization, Wash and Stain Kit (ThermoFisher) according to manufacturer’s instructions. Arrays were scanned in a GeneChip GCS300 7G Scanner and analysed with the Affymetrix Expression Console Software 1.3.1 (ThermoFisher) for the human array and Transcriptome Analysis Console (TAC) 4.0.2 for the mouse arrays. Samples were processed at the Core Facility Molecular Biology at the Centre of Medical Research at the Medical University of Graz, Austria. Pre-processing of CEL-files for the human arrays was performed with Partek Genomics Suite version 6.6 (Partek Inc, St Louis, MO, USA) using the robust multi-chip analysis (RMA) algorithm, which includes background adjustment, quantile normalisation and median polished probe summarisation. For statistical analysis, paired-sample t-tests were used and genes with p<0.05 and a fold change of at least 1.5 were considered to be significantly deregulated. ﻿All microarray data has been deposited at the public repository Gene Expression Omnibus (GEO) (http://www.ncbi.nlm.nih.gov/geo/) with accession numbers GSE145126 and GSE145127.

For analysis of histological responders compared to non-responders, as well as mouse arrays, pre-processing and RMA normalization was done with RStudio Version 1.2.1335 (R Version 3.5.1) with MicroArrayPipeline v1.0 Shiny app based on limma Bioconductor package for differential expression analysis and genes with p<0.05 and a fold change of at least 1.5 were considered to be significantly deregulated. Mouse and human DEGs were tested for potential overlap. Probeset IDs of early (day 6) as well as late (week 2–3) DEGs of human microarray were matched with DEGs of mouse microarray using NetAffxTM Expression Array Comparison Tool.

#### Nanostring nCounter analysis and microarray verification

For Nanostring analysis, a nCounter GX Custom codeset (80 target genes, four reference genes, see Supplementary file 2) was designed to verify microarray results of selected DEGs. Total RNA (150 ng) with RIN values between 4.7 and 9 was used and samples were processed according to supplier’s instructions (NanoString Technologies, Seattle, WA USA) and scanned on a nCounter Digital Analyzer (NanoString Technologies). RCC files were used for data analysis. Raw data pre-processing and normalization was performed using nSolver 2.5 Software (NanoString Technologies) according to standard procedures (background subtraction, positive and negative controls normalization). Gene counts were then normalized to the geometric mean of the reference genes. Normalized data was uploaded to Partek Genomic Suite Software v6.6 (Partek Inc, St Louis, MO, USA) and paired t-test was used for statistical analysis. Nanostring and microarray fold change values of selected target genes were log-transformed and the two platforms were compared by Pearson correlation of each gene across samples.

#### Gene ontology enrichment analysis

For all comparisons genes with a p-value<0.05 and FC ± 1.5 were assigned to GO enrichment analysis using Cytoscape software ver.3.5.1 (Cytoscape Consortium, NY, USA www.cytoscape.org; Bindea et al., 2009). Gene identifiers were loaded into Cytoscape software and ClueGO analysis was used to identify significantly overrepresented GO terms and associated genes. P-values (significance level <0.05) were adjusted using Bonferroni step-down corrections.

#### RT-qPCR

Per sample 2 µg of RNA was reverse transcribed into cDNA using iScript Reverse Transcription Supermix (Bio-Rad, Hercules, CA, USA). Relative gene expression was determined using GoTag qPCR Master Mix (Promega, Mannheim, Germany) on a CFX96 Touch Real-Time PCR Detection System (Bio-Rad). The following cycling conditions were used: Hot-start activation (95°C, 2 min), denaturation for 40 cycles (95°C, 15 s) and annealing/extension (60°C, 60 s). Melting curve analysis was done to confirm amplification specificity. For each sample, qPCRs were run in triplicates. Cycle thresholds (Ct) were determined and relative mRNA expression to *Ubc or Ywhaz* (reference genes) were calculated using the ΔCt method. Primer sequences are listed in Supplementary file 4.

### Animals

#### Mouse strains and housing

6–9 week-old mice were kept with food and water ad libitum in the conventional animal facility at the Centre for Medical Research, Medical University of Graz or at the Medical University of Vienna, Austria. During experiments, all mice were monitored closely to ensure sufficient health status. BALB/c mice were purchased from Charles River (Sulzfeld, Germany). c-Jun/JunB knockout mice (Zenz et al., 2005) were bred and maintained in the facilities of the Medical University of Vienna. All animal experiments were in accordance with institutional policies and federal guidelines.

#### Therapeutic agents used in mice

For all animal experiments, dithranol in different concentrations (dissolved in vaseline) and vehicle (vaseline cream only) was provided by the pharmacy of the Medical University of Graz, Austria. Aldara (IMQ) 5% cream (MEDA Pharmaceuticals, Vienna, Austria) and tamoxifen (Sigma-Aldrich, Missouri, USA) were purchased.

#### Imiquimod model

24 hr before starting an experiment, dorsal skin of BALB/c mice was shaved carefully. To induce psoriasis-like dermatitis, imiquimod (IMQ) cream was applied daily for five consecutive days on dorsal skin (approximately 40 mg) and right ear (approximately 20 mg). A daily topical dose of 62.5 mg of IMQ cream was not exceeded; translating into 3.125 mg of the active compound. Mice received IMQ cream in the morning and dithranol treatment in the afternoon, with a time gap of 8 hr. Dithranol was applied topically on dorsal skin (40 mg) and right ear (20 mg) and concentrations were increased every other day (0.01% on day 1–2, 0.03% on day 3–4% and 0.1% on day 5–6). Control mice were treated similarly with Vaseline cream. Double skin fold of dorsal skin and ear thickness was measured daily in triplicates before any application using a micrometer (Mitutoyo, Kanagawa, Japan). 24 hr after the last topical treatment, mice were sacrificed and approximately 1 cm^2^ of dorsal skin, both ears (treated and untreated as control) were collected.

#### c-Jun/JunB knockout mouse model

Mice carrying floxed alleles for the JunB and c-Jun locus and expressing the K5-CreERT transgene (mixed background) received consecutive i.p. injections of tamoxifen (1 mg/day) for a period of 5 days, leading to deletion of both genes in the epidermis by inducible Cre-recombinase activity (Schonthaler et al., 2009; Zenz et al., 2005). One week after the last injection, psoriasis-like lesions on the ears were treated daily with dithranol in series of increasing concentrations (0.01% on day 1–2, 0.03% on day 3–4% and 0.1% on day 5–6 or 0.1% on day 1–2, 0.3% on day 3–4% and 1% on day 5–6) as depicted in Figure 3A. Control mice were treated similarly with vehicle cream. Ear thickness was measured daily by micrometer before any topical application and 24 hr after the last topical treatment mice were sacrificed and ears were collected.

#### Mouse tail test

For the mouse-tail model, 40 mg dithranol 1% was applied daily for 14 days to the surface of the proximal part of tails (approx. 2 cm in length starting 1 cm from the body). 24 hr after the last application, mice were sacrificed, and treated parts of tail skin were removed from the underlying cartilage. The mouse-tail test for psoriasis is a traditional model to quantify the effect of topical anti-psoriatics on keratinocyte differentiation by measuring degree of orthokeratosis versus parakeratosis (Bosman et al., 1992; Sebök et al., 2000; Wu et al., 2015).

### Statistical analyses

Statistical analyses for human and murine microarrays were performed as described in the specific sections. All other statistical analyses were determined using GraphPad Prism version 8 (GraphPad software, California, USA). Normality was determined by Shapiro-Wilk test and differences between two groups were assessed by Mann Whitney test, Wilcoxon test or T-test (paired or unpaired) as appropriate. For multiple comparisons, One-way ANOVA with Dunnett’s test or Tukey’s test was used for parametric data and Kruskal Wallis test with Dunn’s test was used for nonparametrical data as indicated in the specific sections. Significance was set at a p-value of ≤0.05. Each animal experiment was performed at least twice. For correlation analysis of clinical scores, as well as comparison of microarray and nanostring ratios, Pearson or Spearman correlation was used as indicated.

### Study approval

A clinical study (Clinical Trials.gov no. NCT02752672) in psoriatic patients treated with dithranol was completed in cooperation with the Department of Dermatology, Klagenfurt State Hospital. Clinical trial procedures were approved by the ethics committee of the federal state of Carinthia, Austria (protocol number A23/15) and all participants gave written informed consent in accordance with the principles of the Declaration of Helsinki. All mouse experiments were approved by the Austrian Government, Federal Ministry for Science and Research (protocol numbers BMWF-66-010/0032-11/3b/2018, 66.009/0200-WF/II/3b/2014) and animal experiments performed in Vienna were additionally approved by the Animal Experimental Ethics Committee of the Medical University of Vienna.

## Data Availability

All microarray data has been deposited at the public repository Gene Expression Omnibus (GEO) (http://www.ncbi.nlm.nih.gov/geo/) with accession numbers GSE145126 and GSE145127. The following datasets were generated: BenezederTPainsiCPatraVDeySHolcmannMLange-AsschenfeldtBSibiliaMWolfP2020Microarray analysis of c-Jun/JunB knockout mice treated with dithranolNCBI Gene Expression OmnibusGSE145126 BenezederTPainsiCPatraVDeySHolcmannMLange-AsschenfeldtBSibiliaMWolfP2020Microarray analysis of dithranol-treated psoriasisNCBI Gene Expression OmnibusGSE14512710.7554/eLife.56991PMC726664132484435

## Additional files

Supplementary file 1. Top 45 upregulated genes in lesional psoriatic skin compared to non-lesional skin at baseline from 15 patients with psoriasis (p<0.05, fold change >1.5).

Supplementary file 2. Verification of microarray results of dithranol-treated skin samples of psoriasis patients with Nanostring analysis (nCounter GX Custom codeset) of 80 target genes and four reference genes. FC, fold change

Supplementary file 3. Top 20 significantly enriched pathways as determined by Gene Ontology (GO) enrichment analysis in histological responders compared to non-responders in the psoriasis patient cohort (GO was done using Cytoscape software; [Bindea et al., 2009; Shannon et al., 2003] a.o. = among others).

Supplementary file 4. qPCR Primer sequences and corresponding annealing temperatures.

Transparent reporting form
