# Supplementary material for: Dithranol targets keratinocytes, their crosstalk with neutrophils and inhibits the IL-36 inflammatory loop in psoriasis
Source: eLife. 2020 Jun 2;9:e56991. doi: 10.7554/eLife.56991 (PMC7266641; doi:10.7554/eLife.56991)
Supplement: Supplementary file 1. [file elife-56991-supp1.docx]

**Supplementary File 1:** Top 45 upregulated genes in lesional psoriatic skin compared to non-lesional skin at baseline from 15 patients with psoriasis (P<0.05, fold change >1.5).

| Probe set ID | Gene Symbol | Gene title | Lesional skin vs. Non-lesional skin (fold change) |
| --- | --- | --- | --- |
| 16693375 | SPRR2F | Small proline-rich protein 2F | 144,52 |
| 16671144 | S100A7A | S100 calcium binding protein A7A | 133,99 |
| 17074361 | DEFB4B | Defensin, beta 4B | 113,44 |
| 16738803 | TCN1 | Transcobalamin I (vitamin B12 binding protein, R binder family) | 83,47 |
| 16730157 | HEPHL1 | Hephaestin-like 1 | 60,80 |
| 16693409 | S100A12 | S100 calcium binding protein A12 | 59,88 |
| 16693365 | SPRR2C | Small proline-rich protein 2C | 52,74 |
| 16671139 | S100A9 | S100 calcium binding protein A9 | 52,11 |
| 16976438 | TMPRSS11D | Transmembrane protease, serine 11D | 36,11 |
| 16884602 | IL36A | Interleukin 36, alpha | 32,83 |
| 16967771 | CXCL8 | Chemokine (C-X-C motif) ligand 8 | 29,59 |
| 16693339 | LCE3A | Late cornified envelope 3A | 28,89 |
| 16693331 | LCE3E | Late cornified envelope 3E | 23,31 |
| 16813112 | RHCG | Rh family, C glycoprotein | 22,97 |
| 16773260 | ATP12A | ATPase, H+/K+ transporting, nongastric, alpha polypeptide | 20,70 |
| 16693414 | S100A8 | S100 calcium binding protein A8 | 20,61 |
| 17051827 | AKR1B10 | Aldo-keto reductase family 1, member B10 | 16,23 |
| 16807797 | PLA2G4D | Phospholipase A2 | 16,15 |
| 17089525 | LCN2 | Lipocalin 2 | 15,90 |
| 16967831 | EPGN | Epithelial mitogen | 15,74 |
| 16979444 | TNIP3 | TNFAIP3 interacting protein 3 | 15,69 |
| 16861914 | PAPL | Iron/zinc purple acid phosphatase-like protein | 15,67 |
| 16886174 | KYNU | Kynureninase | 15,26 |
| 16777185 | GJB2 | Gap junction protein, beta 2, 26kDa | 15,00 |
| 17085829 | GDA | Guanine deaminase | 14,82 |
| 17065453 | DEFB103A | Defensin, beta 103A | 14,62 |
| 16844817 | KRT16 | Keratin 16 | 13,41 |
| 17106398 | SLC6A14 | Solute carrier family 6 (amino acid transporter), member 14 | 13,15 |
| 16977052 | CXCL10 | Chemokine (C-X-C motif) ligand 10 | 11,82 |
| 17083357 | CD274 | CD274 molecule | 10,67 |
| 17023716 | VNN3 | Vanin 3 | 9,51 |
| 16777190 | GJB6 | Gap junction protein, beta 6 | 8,77 |
| 17066961 | ADAMDEC1 | ADAM-like, decysin 1 | 8,04 |
| 16901986 | IL1B | Interleukin 1, beta | 7,64 |
| 16668622 | CHI3L2 | Chitinase 3-like 2 | 7,64 |
| 16799739 | CHAC1 | Cation transport regulator homolog 1 | 6,78 |
| 16896196 | XDH | Xanthine dehydrogenase | 6,73 |
| 16666943 | GBP6 | Guanylate binding protein family, member 6 | 6,58 |
| 16731773 | TMPRSS4 | Transmembrane protease, serine 4 | 6,56 |
| 16863419 | IGFL1 | IGF-like family member 1 | 6,49 |
| 16693357 | SPRR2A | Small proline-rich protein 2A | 6,28 |
| 17065458 | DEFB4A | Defensin, beta 4A | 6,10 |
| 16675301 | RGS1 | Regulator of G-protein signaling 1 | 5,57 |
| 17059771 | SAMD9 | Sterile alpha motif domain containing 9 | 5,53 |
| 16860418 | CCNE1 | Cyclin E1 | 5,41 |
|  |  |  |  |
