# Supplementary material for: Dithranol targets keratinocytes, their crosstalk with neutrophils and inhibits the IL-36 inflammatory loop in psoriasis
Source: eLife. 2020 Jun 2;9:e56991. doi: 10.7554/eLife.56991 (PMC7266641; doi:10.7554/eLife.56991)
Supplement: Supplementary file 2. — FC, fold change [file elife-56991-supp2.docx]

**Supplementary File 2:** Verification of microarray results of dithranol-treated skin samples of psoriasis patients with Nanostring analysis (nCounter GX Custom codeset) of 80 target genes and 4 reference genes. FC, fold change

|  | Day 6 vs. Baseline | | | | End of treatment vs. Baseline | | | |
| --- | --- | --- | --- | --- | --- | --- | --- | --- |
| Gene symbol | Array  p-value | Array  FC | Nanostring  p-value | Nanostring  FC | Array  p-value | Array  FC | Nanostring  p-value | Nanostring  FC |
| AADAC | 0.0856653 | 1.68 | 0.163549 | 2.07 | 0.0359689 | 3.07 | 0.0414731 | 5.94 |
| AIM2 | 0.560754 | -1.08 | 0.0880365 | -1.33 | 0.00423871 | -1.92 | 0.00059396 | -2.79 |
| BCL2A1 | 0.0309898 | -1.52 | 0.0569415 | -1.79 | 0.0282255 | -2.14 | 0.0101236 | -3.48 |
| BTC | 0.237518 | 1.18 | 0.476442 | 1.48 | 0.0152396 | 2.27 | 0.0116771 | 9 |
| CASP14 | 0.00428852 | 1.68 | 0.0179154 | 1.73 | 0.133415 | 1.64 | 0.0728102 | 1.75 |
| CCL20 | 0.966376 | -1.01 | 0.545059 | -1.19 | 0.0478305 | -2.19 | 0.0263852 | -2.96 |
| CCL21 | 0.872015 | -1.02 | 0.840844 | -1.03 | 8.78E+00 | 1.57 | 0.034687 | 1.37 |
| CCL22 | 0.611391 | -1.09 | 0.189598 | -1.37 | 0.0283274 | -1.66 | 0.00324819 | -2.84 |
| CCR4 | 0.851695 | -1.03 | 0.587396 | -1.1 | 0.0433418 | -1.43 | 0.0567474 | -1.4 |
| CD1B | 0.578657 | 1.15 | 0.621682 | 1.14 | 0.00345005 | -1.93 | 0.0303729 | -2.09 |
| CD1E | 0.130048 | 1.4 | 0.219978 | 1.81 | 0.472134 | -1.17 | 0.723306 | 1.21 |
| CD207 | 0.0302958 | 1.63 | 0.181563 | 1.82 | 0.459015 | 1.38 | 0.557965 | 1.46 |
| CD274 | 0.0739066 | -1.5 | 0.0213586 | -1.98 | 0.00755108 | -3.35 | 0.00250642 | -5.8 |
| CLDN8 | 0.919788 | 1.03 | 0.385784 | 1.86 | 0.0490195 | 2.11 | 0.00808229 | 5.82 |
| CLEC2A | 0.0107815 | 1.92 | 0.00930877 | 1.89 | 0.165193 | 1.66 | 0.0721891 | 1.82 |
| CXCL10 | 0.667388 | -1.09 | 0.313074 | -1.2 | 0.137836 | -1.47 | 0.181068 | -1.63 |
| CXCL13 | 0.0357349 | -1.42 | 0.20209 | -1.69 | 0.00228843 | -2.71 | 0.00231808 | -8.81 |
| CXCL17 | 0.662216 | -1.08 | 0.304704 | -1.47 | 0.0129313 | -1.79 | 0.0010903 | -9.9 |
| CXCL5 | 0.0185656 | -1.81 | 0.16518 | -2.61 | 0.169262 | -1.84 | 0.254181 | -2.62 |
| CXCL8 | 0.0493238 | -3.36 | 0.0476054 | -6.58 | 0.0019015 | -9.14 | 0.00494871 | -48.37 |
| DEFB103A | 0.054042 | -1.52 | 0.979441 | 1 | 0.0112862 | -3.94 | 0.809766 | -1.02 |
| DEFB4A | 0.019896 | -1.75 | 0.164403 | -1.92 | 0.00324037 | -3.01 | 0.00392997 | -23.31 |
| DSC2 | 0.0265007 | -1.31 | 0.0349425 | -1.42 | 0.0199662 | -2.14 | 0.0111283 | -2.72 |
| DSG2 | 0.971063 | -1.01 | 0.793248 | 1.06 | 0.089218 | 1.58 | 0.0567873 | 1.76 |
| ELMOD1 | 0.00974332 | 1.91 | 0.0974323 | 2.5 | 0.12226 | 1.71 | 0.121403 | 2.99 |
| EPGN | 0.117466 | -1.67 | 0.0225424 | -2.28 | 0.0236151 | -5.31 | 0.00571018 | -11.9 |
| FLG | 0.0431728 | 1.97 | 0.0644869 | 2.25 | 0.15329 | 2.15 | 0.0704037 | 3.39 |
| FLG2 | 0.0492031 | 2.89 | 0.0920579 | 3.29 | 0.0957116 | 3.66 | 0.0509036 | 5.74 |
| GJB6 | 0.603224 | -1.06 | 0.528654 | -1.1 | 0.0076714 | -2.12 | 0.00342742 | -2.31 |
| GUSB | 0.677478 | 1.03 | 0.470834 | -1.05 | 0.478471 | -1.06 | 0.739332 | 1.03 |
| HRNR | 0.0341734 | 2.64 | 0.0452725 | 2.52 | 0.463593 | 1.22 | 0.570038 | -1.43 |
| IFNG | 0.474642 | -1.18 | 0.0107162 | -3.53 | 0.00488445 | -1.93 | 6.53E+00 | -9.33 |
| IL17A | 0.0562996 | -1.44 | 0.0252835 | -3.5 | 0.00379919 | -2.14 | 0.00018563 | -14.87 |
| IL1B | 0.113333 | -1.88 | 0.0540175 | -3.28 | 0.0154153 | -3.3 | 0.00124276 | -16.07 |
| IL20 | 0.117439 | -1.74 | 0.01724 | -4.14 | 0.0304691 | -2.82 | 0.00152459 | -37.95 |
| IL22 | 0.256745 | -1.43 | 0.0343073 | -3.71 | 0.00186577 | -3.87 | 0.00307876 | -8.08 |
| IL22RA2 | 0.219132 | 1.24 | 0.237187 | 1.79 | 0.0718037 | -1.31 | 0.125244 | -1.95 |
| IL26 | 0.467642 | -1.08 | 0.0409937 | -1.57 | 0.0114166 | -1.63 | 0.00035757 | -9.4 |
| IL36A | 0.05178 | -2.01 | 0.0399339 | -2.36 | 0.00551108 | -10.5 | 0.00292568 | -25.04 |
| IL36G | 0.19467 | -1.28 | 0.113075 | -1.42 | 0.00736648 | -3.08 | 0.00266324 | -3.72 |
| IL36RN | 0.335566 | -1.19 | 0.136412 | -1.37 | 0.0142878 | -2.31 | 0.0024377 | -2.73 |
| IRAK2 | 0.538699 | -1.12 | 0.423602 | -1.15 | 0.0142498 | -1.77 | 0.0178164 | -1.89 |
| IRF8 | 0.603387 | 1.04 | 0.368949 | 1.11 | 0.00422117 | -1.48 | 0.040135 | -1.31 |
| IVL | 0.557743 | -1.11 | 0.690318 | -1.07 | 0.0271582 | -1.76 | 0.0110917 | -1.9 |
| KIAA1324 | 0.971445 | -1.01 | 0.602521 | -1.12 | 0.120721 | 1.85 | 0.72233 | 1.16 |
| KRT16 | 0.0542839 | -1.25 | 0.993356 | 1 | 0.0126119 | -2.75 | 0.0703736 | -3.84 |
| KRT17 | 0.0655637 | -1.39 | 0.107782 | -1.63 | 0.0243129 | -2.71 | 0.0140719 | -4.74 |
| KRT2 | 0.0182126 | 2.04 | 0.101039 | 1.87 | 0.149695 | 2.17 | 0.0545458 | 3.09 |
| KRT6A | 0.165236 | -1.43 | 0.192393 | -1.43 | 0.0162569 | -3.69 | 0.00661455 | -5.41 |
| KRT73 | 0.0249938 | 1.66 | 0.552553 | 1.37 | 0.0146097 | 2.31 | 0.0655098 | 3.09 |
| LCE1A | 0.0381688 | 1.55 | 0.0655631 | 1.51 | 0.517969 | 1.36 | 0.40037 | 1.46 |
| LCE1C | 0.0167379 | 1.71 | 0.0483371 | 1.6 | 0.330256 | 1.63 | 0.269475 | 1.67 |
| LCE2D | 0.0312116 | 1.55 | 0.0574707 | 1.52 | 0.405432 | 1.49 | 0.457545 | 1.44 |
| LCE3C | 0.447262 | -1.19 | 0.18415 | -1.39 | 0.00992198 | -4.14 | 0.00491206 | -11.2 |
| LCE3E | 0.867951 | 1.03 | 0.921617 | -1.02 | 0.0320675 | -2.27 | 0.0148307 | -2.24 |
| NEB | 0.0138626 | 2.18 | 0.0444461 | 1.74 | 0.0481161 | 1.8 | 0.212716 | 1.35 |
| NOS2 | 0.0661324 | -2.52 | 0.00503528 | -11.28 | 0.00093779 | -5.38 | 0.0001623 | -30.44 |
| PI3 | 0.167827 | -1.13 | 0.212487 | -1.36 | 0.0191005 | -2.84 | 0.00365026 | -6.89 |
| PPBP | 0.0193783 | -1.82 | 0.00030535 | -10.62 | 0.426709 | -1.4 | 0.0397445 | -5.47 |
| PRKCQ | 0.228953 | -1.1 | 0.793119 | 1.02 | 0.0151713 | -2.01 | 0.0551467 | -2.11 |
| PSORS1C2 | 0.166341 | 1.24 | 0.670683 | 1.08 | 0.230892 | -1.26 | 0.0111578 | -1.8 |
| RGS1 | 0.29889 | -1.24 | 0.120906 | -1.46 | 0.00874502 | -2.53 | 0.0130182 | -2.84 |
| RPL13A | 0.186734 | -1.09 | 0.206526 | 1.09 | 0.172996 | -1.09 | 0.780901 | 1.03 |
| RPTN | 0.105776 | 1.59 | 0.0923132 | 1.58 | 0.0451319 | -1.42 | 0.0362379 | -1.5 |
| S100A12 | 0.376792 | -1.2 | 0.452277 | -1.17 | 0.00417422 | -6.12 | 0.00334604 | -6.77 |
| S100A7A | 0.478358 | -1.17 | 0.086662 | -1.66 | 0.0102531 | -5 | 0.00286694 | -8.26 |
| S100A8 | 0.25983 | -1.07 | 0.198151 | -1.18 | 0.0127109 | -1.85 | 0.00381322 | -3.32 |
| S100A9 | 0.390127 | -1.08 | 0.284456 | -1.17 | 0.0187374 | -2.12 | 0.0029869 | -3.09 |
| SEMA3E | 0.809239 | -1.08 | 0.763916 | -1.12 | 0.213343 | 1.44 | 0.584314 | 1.3 |
| SEMA7A | 0.858271 | 1.02 | 0.630293 | -1.1 | 0.019966 | -1.48 | 0.00379147 | -1.99 |
| SERPINB1 | 0.00623796 | -1.67 | 0.00409089 | -1.84 | 0.0210101 | -2.17 | 0.00926207 | -2.5 |
| SERPINB12 | 0.0459936 | 1.74 | 0.0967613 | 1.7 | 0.516108 | 1.36 | 0.438333 | 1.44 |
| SERPINB9 | 0.11554 | -1.25 | 0.138688 | -1.28 | 0.00888896 | -2.2 | 0.00315224 | -2.2 |
| SLC46A2 | 0.0225234 | 1.43 | 0.0891011 | 3.06 | 0.028197 | 2.07 | 0.0410747 | 7.85 |
| SLC6A14 | 0.0269294 | -1.69 | 0.0273005 | -1.92 | 0.00507889 | -4.6 | 0.00167204 | -6.41 |
| SPRR2D | 0.161959 | -1.4 | 0.173765 | -1.32 | 0.0197032 | -2.38 | 0.00576619 | -3.28 |
| SPRR2F | 0.0483771 | -1.69 | 0.0489185 | -1.95 | 0.00637096 | -10.12 | 0.00576253 | -17.56 |
| TCN1 | 0.267823 | -1.17 | 0.43935 | -1.12 | 0.00186777 | -6.45 | 0.00167819 | -6.15 |
| TGM1 | 0.192932 | -1.28 | 0.172113 | -1.26 | 0.00962934 | -2.39 | 0.00342298 | -2.52 |
| TNFRSF12A | 0.00489713 | -1.6 | 0.0119923 | -2.84 | 0.0251288 | -1.8 | 0.0142822 | -3.75 |
| TNIP3 | 0.0857776 | -1.81 | 0.00749388 | -2.86 | 0.0120739 | -4.48 | 0.0008823 | -40.61 |
| TREM1 | 0.0472953 | -1.99 | 0.069627 | -5.19 | 0.0254955 | -2.13 | 0.00784702 | -7.34 |
| TUBB | 0.574093 | -1.03 | 0.830364 | 1.01 | 0.126295 | 1.1 | 0.0421854 | 1.13 |
| YWHAZ | 0.359763 | -1.02 | 0.434349 | -1.05 | 0.273065 | -1.05 | 0.0346371 | -1.2 |
|  |  |  |  |  |  |  |  |  |
