# Supplementary material for: Dithranol targets keratinocytes, their crosstalk with neutrophils and inhibits the IL-36 inflammatory loop in psoriasis
Source: eLife. 2020 Jun 2;9:e56991. doi: 10.7554/eLife.56991 (PMC7266641; doi:10.7554/eLife.56991)
Supplement: Supplementary file 3. [file elife-56991-supp3.docx]

**Supplementary File 3:** Top 20 significantly enriched pathways as determined by Gene Ontology (GO) enrichment analysis in histological responders compared to non-responders in the psoriasis patient cohort (GO was done using Cytoscape software; (Bindea et al., 2009; Shannon et al., 2003) a.o. = among others)

| **GO ID** | **GO Term** | **P-Value** | **% Associated Genes** | **Associated Genes Found** |
| --- | --- | --- | --- | --- |
| GO:0030216 | Keratinocyte differentiation | 89,0E-33 | 8,05 | DSG4, KRT16, KRT23, KRT32, KRT33A, LCE2B, LCE2C, LCE5A, WNT5A, a.o. |
| GO:0005882 | Intermediate filament | 400,0E-30 | 9,57 | KRT16, KRT82, KRTAP19-1, KRTAP19-5, KRTAP2-4, KRTAP29-1, a.o. |
| GO:0007925 | Formation of hair keratin fibres | 2,4E-27 | 12,16 | KRT23, KRT32, KRT33A, KRT35, KRTAP2-4, KRTAP29-1, KRTAP3-3, a.o. |
| GO:0045095 | Keratin filament | 1,8E-18 | 11,88 | KRT82, KRTAP2-4, KRTAP29-1, KRTAP3-3, KRTAP4-1, KRTAP4-12, a.o. |
| GO:0007874 | Keratin filament formation | 950,0E-12 | 7,29 | DSG4, KRT16, KRT23, KRT32, KRT33A, KRT35, KRT82 |
| GO:0007875 | Keratin tetramers bind to form unit length filaments | 950,0E-12 | 7,29 | DSG4, KRT16, KRT23, KRT32, KRT33A, KRT35, KRT82 |
| GO:0007876 | Formation of tonofilament bundles | 950,0E-12 | 7,29 | DSG4, KRT16, KRT23, KRT32, KRT33A, KRT35, KRT82 |
| GO:0007923 | Keratin filaments bind cell-cell adhesion complexes | 950,0E-12 | 7,29 | DSG4, KRT16, KRT23, KRT32, KRT33A, KRT35, KRT82 |
| GO:0007950 | Lamellar bodies bind the early cornified envelope | 950,0E-12 | 7,29 | DSG4, KRT16, KRT23, KRT32, KRT33A, KRT35, KRT82 |
| GO:0007972 | Reinforcement of the Cornified Envelope | 950,0E-12 | 7,29 | DSG4, KRT16, KRT23, KRT32, KRT33A, KRT35, KRT82 |
| GO:0008011 | CDSN binds the cornified envelope | 950,0E-12 | 7,29 | DSG4, KRT16, KRT23, KRT32, KRT33A, KRT35, KRT82 |
| GO:0009258 | Filaggrin binds Keratin tonofilament:Desmosome | 950,0E-12 | 7,29 | DSG4, KRT16, KRT23, KRT32, KRT33A, KRT35, KRT82 |
| GO:0070268 | Cornification | 2,9E-9 | 6,19 | DSG4, KRT16, KRT23, KRT32, KRT33A, KRT35, KRT82 |
| GO:0042633 | Hair cycle | 3,2E-6 | 4,39 | DSG4, KRT16, KRTAP4-7, KRTAP4-8, WNT5A |
| GO:0000393 | Antigen processing by cathepsin S in endosoytic vesicle | 290,0E-6 | 4,62 | CTSH, HLA-B, HLA-DQB2 |
| GO:0001372 | TLR9 processing at neutral pH | 290,0E-6 | 4,62 | CTSH, HLA-B, HLA-DQB2 |
| GO:0002937 | Generation of CLIP from lip10 | 290,0E-6 | 4,62 | CTSH, HLA-B, HLA-DQB2 |
| GO:0002943 | Cleavage of lip22 to lip10 | 290,0E-6 | 4,62 | CTSH, HLA-B, HLA-DQB2 |
| GO:0002947 | MHC class II antigen processing | 290,0E-6 | 4,62 | CTSH, HLA-B, HLA-DQB2 |
| GO:0003462 | Fibrillin-1 degradation by MMP3, CTSK, CTSL2 | 290,0E-6 | 4,62 | CTSH, HLA-B, HLA-DQB2 |
|  |  |  |  |  |
