# Supplementary material for: Dithranol targets keratinocytes, their crosstalk with neutrophils and inhibits the IL-36 inflammatory loop in psoriasis
Source: eLife. 2020 Jun 2;9:e56991. doi: 10.7554/eLife.56991 (PMC7266641; doi:10.7554/eLife.56991)
Supplement: Supplementary file 4. [file elife-56991-supp4.docx]

Supplementary File 4: qPCR Primer sequences and corresponding annealing temperatures.

|  |  |  |
| --- | --- | --- |
| **Primer ID** | **Sequence** | **Annealing Temperature (°C)** |
| Cxcl1 | Fw: ATCCAGAGCTTGAAGGTGTTG; | 60 |
|  | Rev: GTCTGTCTTCTTTCTCCGTTACTT |  |
| Cxcl5 | Fw: CTCAGTCATAGCCGCAACCGAGC; | 60 |
|  | Rev: CGCTTCTTTCCACTGCGAGTGC |  |
| Defb1 (Defensin beta 1) | Fw: CCAGCTGCCCATCTAATACC; | 60 |
|  | Rev: AATCCATCGCTCGTCCTTTA |  |
| Defb3 (Defensin beta 3) | Fw: TCTGACGAGTGTTGCCAATG; | 60 |
|  | Rev: ACAATCCAGTAAGTTGTTTGAGGA |  |
| Flg | Fw: GAAGGAACTTCTGGAAGGACAAC; | 60 |
|  | Rev: TCCATCAGTTCCACCATGCCTC |  |
| Il17 | Fw: GGACTCTCCA CCGCAATGA; | 60 |
|  | Rev: TCAGGCTCCCTCTTCAGGAC |  |
| Il1b | Fw: GAGTGTGGATCCCAAGCAAT; | 58 |
|  | Rev: TACCAGTTGGGGAACTCTGC |  |
| Il1f5 (IL36RN) | Fw: CTTACTCCTCTCCTTCCCTACT; | 60 |
|  | Rev: GGTCATCTTCTGTCAGCTATCC |  |
| Il22 | Fw: CAGCTCCTGTCACATCAGCGGT; | 60 |
|  | Rev: AGGTCCAGTTCCCCAATCGCCT |  |
| Ivl | Fw: AAACTTGGTGAGCCAGAATTACA; | 60 |
|  | Rev: CCTTTCCAGTTGTTTACCCTTCT |  |
| Krt16 | Fw: AGCAGGAGATCGCCACCTA; | 60 |
|  | Rev: AGTGCTGTGAGGAGGAGTGG |  |
| Lce3e | Fw: GCCCTGCTGACTTCTTCTATCCAG; | 63 |
|  | Rev: AGCTACCAGGGAATGAGGACTGTG |  |
| Lcn2 | Fw: CCCTGTATGGAAGAACCAAGGA; | 60 |
|  | Rev: CACACTCACCACCCATTCAGT |  |
| LL37 (Camp) | Fw: CTTCAAGGAACAGGGGGTG; | 60 |
|  | Rev: CCAAGGCAGGCCTACTACTC |  |
| Rpl13a | Fw: CACTCTGGAGGAGAAACGGAAGG; | 63 |
|  | Rev: GCAGGCATGAGGCAAACAGTC |  |
| S100A8 | Fw: AAATCACCATGCCCTCTACAAG; | 58 |
|  | Rev: CCCACTTTTATCACCATCGCAA |  |
| S100A9 | Fw: GGTGGAAGCACAGTTGGCA; | 58 |
|  | Rev: GTGTCCAGGTCCTCCATGATG |  |
| Serpbinb7 | Fw: CTTCTCTTCCCTGAGCATCTTC; | 60 |
|  | Rev: GTGAAGGCCGATTTCCATTTG |  |
| Serpinb13 | Fw: CCCAGTTCAACCTGGAAGATAG; | 60 |
|  | Rev: CCAGAGTAGATCAAGGGCAATAG |  |
| Serpinb3a | Fw: TCCTGTTGCCAGTGGAAATCA; | 60 |
|  | Rev: TCAAAGGCATCGACCATTCCC |  |
| Ubc | Fw: AGGTCAAACAGGAAGACAGACGTA; | 60 |
|  | Rev: TCACACCCAAGAACAAGCACA |  |
| Ywhaz | Fw: AACAGCTTTCGATGAAGCCAT; | 60 |
|  | Rev: TGGGTATCCGATGTCCACA |  |
|  |  |  |
